# Roles of oral microbiota and oral-gut microbial transmission in hypertension

**DOI:** 10.1016/j.jare.2022.03.007

**Published:** 2022-03-19

**Authors:** Bo-Yan Chen, Wen-Zhen Lin, Yu-Lin Li, Chao Bi, Lin-Juan Du, Yuan Liu, Lu-Jun Zhou, Ting Liu, Shuo Xu, Chao-Ji Shi, Hong Zhu, Yong-Li Wang, Jian-Yong Sun, Yan Liu, Wu-Chang Zhang, Hai-Xia Lu, Yi-Hua Wang, Qiang Feng, Fu-Xiang Chen, Chang-Qian Wang, Maurizio S. Tonetti, Ya-Qin Zhu, Huili Zhang, Sheng-Zhong Duan

**Affiliations:** a Laboratory of Oral Microbiota and Systemic Diseases, Shanghai Ninth People's Hospital, College of Stomatology, Shanghai Jiao Tong University School of Medicine, Shanghai, China; b National Center for Stomatology; National Clinical Research Center for Oral Diseases; Shanghai Key Laboratory of Stomatology, Shanghai, China; c Department of General Dentistry, Shanghai Ninth People’s Hospital, Shanghai Jiao Tong University School of Medicine, Shanghai, China; d Department of Stomatology, First Affiliated Hospital, Anhui Medical University, Hefei, China; e Department of Oral and Maxillofacial-Head and Neck Oncology, Shanghai Ninth People's Hospital, Shanghai Jiao Tong University School of Medicine, Shanghai, China; f Department of Preventive Dentistry, Shanghai Ninth People’s Hospital, College of Stomatology, Shanghai Jiao Tong University School of Medicine, Shanghai, China; g Department of Human Microbiome, School and Hospital of Stomatology, Cheeloo College of Medicine, Shandong University & Shandong Key Laboratory of Oral Tissue Regeneration & Shandong Engineering Laboratory for Dental Materials and Oral Tissue Regeneration, Jinan, China; h Department of Clinical Immunology, Shanghai Ninth People's Hospital, Shanghai Jiao Tong University School of Medicine, Shanghai, China; i Department of Cardiology, Shanghai Ninth People’s Hospital, Shanghai Jiao Tong University School of Medicine, Shanghai, China; j Department of Oral and Maxillofacial Implantology, Shanghai Ninth People’s Hospital, Shanghai Jiao Tong University School of Medicine, Shanghai, China

**Keywords:** Oral microbiota, Gut microbiota, Oral-gut axis, Blood pressure, Hypertension, *Veillonella*, HTN, hypertension, PD, periodontitis, BP, blood pressure, SBP, systolic blood pressure, DBP, diastolic blood pressure, PCoA, Principal coordinate analysis, LEfSe, Linear discriminant analysis effect size, IL-6, Interleukin-6, SNV, single nucleotide variant, ABX, antibiotics cocktail, ANG II, angiotensin II, CRP, C-reactive protein

## Abstract

**Introduction:**

Considerable evidence has linked periodontitis (PD) to hypertension (HTN), but the nature behind this connection is unclear. Dysbiosis of oral microbiota leading to PD is known to aggravate different systematic diseases, but the alteration of oral microbiota in HTN and their impacts on blood pressure (BP) remains to be discovered.

**Objectives:**

To characterize the alterations of oral and gut microbiota and their roles in HTN.

**Methods:**

We performed a cross-sectional (95 HTN participants and 39 controls) and a 6-month follow-up study (52 HTN participants and 26 controls) to analyze the roles of oral and gut microbiota in HTN. Saliva, subgingival plaques, and feces were collected for 16S rRNA gene sequencing or metagenomic analysis. C57BL/6J mice were pretreated with antibiotics to deplete gut microbiota, and then transplanted with human saliva by gavage to test the impacts of abnormal oral-gut microbial transmission on HTN.

**Results:**

BP in participants with PD was higher than no PD in both cross-sectional and follow-up cohort. Relative abundances of 14 salivary genera, 15 subgingival genera and 10 gut genera significantly altered in HTN and those of 7 salivary genera, 12 subgingival genera and 6 gut genera significantly correlated with BP. Sixteen species under 5 genera were identified as oral-gut transmitters, illustrating the presence of oral-gut microbial transmission in HTN. *Veillonella* was a frequent oral-gut transmitter stably enriched in HTN participants of both cross-sectional and follow-up cohorts. Saliva from HTN participants increased BP in hypertensive mice. Human saliva-derived *Veillonella* successfully colonized in mouse gut, more abundantly under HTN condition.

**Conclusions:**

PD and oral microbiota are strongly associated with HTN, likely through oral-gut transmission of microbes. Ectopic colonization of saliva-derived *Veillonella* in the gut may aggravate HTN. Therefore, precise manipulations of oral microbiota and/or oral-gut microbial transmission may be useful strategies for better prevention and treatment of HTN.

## Highlights

• This study has provided comprehensive evidence to support strong associations between oral microbiota and HTN. We discovered that 14 salivary genera and 15 subgingival genera were significantly altered in hypertension participants.
• Fifteen oral genera were first found to associate with blood pressure. There were also new associations between oral microbiota and other clinical parameters been established.
• This study has identified the shared genera that coexisted in saliva, subgingival plaques, and feces and illustrated the associations of the shared genera between oral and gut sample types.
• This study has identified important oral-gut transmitting microbes in hypertension. Sixteen species under 5 genera were identified as oral-gut transmitters. Particularly, *Veillonella* was identified as a frequent oral-gut transmitter stably enriched in hypertension participants.
• This study has demonstrated the potential causal link between oral-gut microbial transmission and hypertension—saliva from participants with hypertension exacerbated angiotensin II-induced hypertension in animal study. *Veillonella* could colonize in the gut of all mice receiving human saliva and more enriched in mice receiving saliva from hypertension participants.

## Graphical abstract

**Figure:**
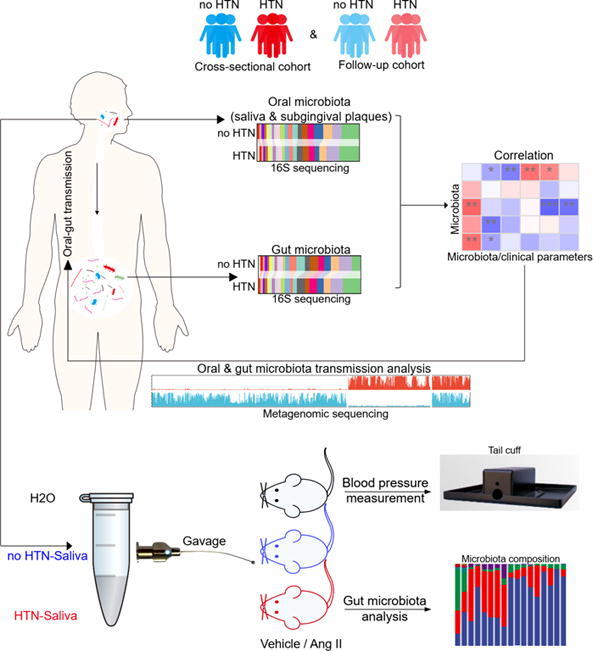

## Introduction

As a global public health concern, hypertension (HTN) is a significant risk factor for cardiovascular diseases and increases all-cause morbidity and mortality worldwide[1]. Although plenty of efforts have been made in investigating the epidemiology and pathophysiology, the etiology of HTN remains complex[2]. Accumulating evidence has indicated gut microbiota as an essential environmental factor involved in the development and progression of HTN[3]. However, it is much less clear whether other microbial flora, for example, oral microbiota, also plays a role in HTN, although the oral cavity is the second largest microbial habitat in human bodies[4].

In fact, very little is known regarding the function of oral microbiota in HTN, although the association between periodontitis (PD) - a common periodontal disease closely related to oral microbial dysbiosis - and HTN has been established. Cross-sectional studies have demonstrated that patients with PD have higher blood pressure (BP) and that more elevated BP usually occurs in patients with more severe PD[5]. Intensive periodontal treatment of PD has been shown to decrease BP more effectively than control periodontal treatment in a randomized controlled trial[6]. Interestingly, the BP reduction correlates with the decrease of periodontal pocket depth, an important indicator for improvement of periodontal status, implying a causal relationship between PD and HTN[6]. Growing evidence has suggested that dysbiotic oral microbiota not only causes PD by undermining periodontal supporting tissues but is also adverse to systematic diseases[7]. A study in older women has suggested associations between oral microbiota and BP[8]. However, the function of oral microbiota dysbiosis in HTN has remained incompletely understood.

The importance of oral-gut microbial transmission in systematic diseases has been increasingly appreciated. Unlike the conventional view considering translocations of oral microbes to the gut rare events because of physiological segregation, recent data indicate that it is common and extensive for oral microbes to translocate to and then colonize in the intestine and that the oral cavity is regarded as an endogenous reservoir for gut microbiota[9]. Moreover, elevated ectopic colonization of oral microbes in the gut has been linked to various disease conditions, including cirrhosis[10], rheumatoid arthritis[11], inflammatory bowel disease[12], Alzheimer's Disease,[13] and even coronavirus disease 2019[14]. However, it is unknown whether oral-gut microbial transmission plays a role in HTN.

In this study, we set out to explore the functions of oral/gut microbiota and oral-gut microbial transmission in HTN. First, we studied the relationship between PD and HTN in both cross-sectional and follow-up cohorts. We also investigated the alterations of oral and gut microbiota in the cross-sectional cohort using 16S rRNA gene sequencing; explored the correlations between oral/gut microbiota and BP/other clinical parameters; surveyed communications between oral and gut microbiota at the genus level. We further analyzed the oral/gut microbiota of this cohort using metagenomic sequencing and explored the oral-gut microbial transmission at the species level. Subsequently, we studied the alterations of oral/gut microbiota and oral-gut microbial transmission in the follow-up cohort. Finally, we investigated the causal link between oral-gut microbial transmission and HTN in mice.

## Materials and methods

Detailed methods are available in the Supplementary Material. Raw sequences are available in the Sequence Read Archive of NIH with accession numbers PRJNA764503, PRJNA765566 and PRJNA774166.

### Ethics statement

All experiments involving human and animals were conducted according to the ethical policies and procedures approved by the Institutional Review and Ethics Board of Shanghai Ninth People’s Hospital, Shanghai Jiao Tong University School of Medicine (Approval no. SH9H-2018-T66-3).

### Study cohorts

In the cross-sectional cohort, 95 participants with HTN and 39 controls without HTN were recruited. A follow-up cohort consisted of 52 HTN participants and 26 controls. HTN diagnosis was based on the criteria of 2018 ESC/ESH Guidelines[2], and periodontitis (PD) was defined according to the gold standard of Centers for Disease Control and Prevention/American Academy of Periodontology case definitions[15]. The detailed criteria for diagnosis of HTN were systolic blood pressure (SBP) ≥ 140 mmHg and/or diastolic blood pressure (DBP) ≥ 90 mmHg. BP of each participant was measured for 3 times with 5-min intervals by a physician using Omron electronic sphygmomanometer, and the average was recorded. The detailed criteria for diagnosis of PD were at least 2 interproximal sites had clinical attachment loss (CAL) ≥ 3 mm and probing depth (PD) ≥ 4 mm. Participants with any of these conditions were excluded: 1. Pregnancy; 2. Smoking; 3. Diseases including peripheral artery disease, autoimmune disease, heart failure, renal failure, cancer, irritable bowel syndrome, inflammatory bowel disease, and recurrent aphthous oral ulcers; 4. Being treated with antibiotics or probiotics, or undergone oral/gut surgeries/treatments within the last 2 months; 5. Having<8 natural teeth. All medical data were collected according to standard clinical procedures. The HTN group had hypertension for an average of 12.3 years. There was no change in medications during the 6-month follow-up period. The body weights of the participants in the follow-up did not change significantly either.

### Sample size calculation

Sample size was estimated using a previously established method[16]. To achieve a minimum correlation coefficient of 0.25 (r = 0.25)[17], 5% significance level (α = 0.05), and 80% test power (β = 0.2) in correlation analyses, a sample size of 123 was required.

### Animal experiments

Ten-week-old male C57BL/6J mice were pretreated with antibiotics cocktail (ABX) to deplete gut microbiota and then treated with sterilized water, saliva from participants with or without HTN by oral gavage. The mice were then subcutaneously implanted with minipumps containing vehicle (saline) or angiotensin II (Ang II). BP was measured using Tail-cuff.

### Microbiota sequencing

Oral and gut samples were collected and DNA was extracted. Both 16S rRNA gene sequencing and metagenomic sequencing were performed at Personal Bio Inc. (Shanghai, China) using Illumina platforms.

### Statistical analysis

Student’s *t*-test, two-way ANOVA followed by Sidak’s multiple comparison test, and Mann-Whitney U rank-sum (MW) test were performed using Prism (GraphPad Software). Kruskal-Wallis rank-sum test and Permutational multivariate analysis of variance were used to analyze alpha diversity and Principal coordinate analysis (PCoA) distance matrix respectively by R software (Version 4.0.2). The Pearson's chi-squared test was used for statistical analysis of sex, drinkers, and follow-up rate between no HTN and HTN. Adonis test was also performed by R software. Kruskal-Wallis rank-sum test was used to analyze the abundance of microbiota. Spearman’s correlations among microbiota, clinic parameters and metabolites were tested and visualized by corrplot R package. Mann-Whitney *U* test and Wilcoxon rank-sum test were performed to test the single nucleotide variant (SNV) distance of oral-gut sample pair between inter-individual and intra-individual. The Linear discriminant analysis effect size was used to identify different taxa/pathway enrichment between no HTN and HTN.

## Results

### PD is associated with higher BP in both cross-sectional and follow-up study

**Fig. 1:**
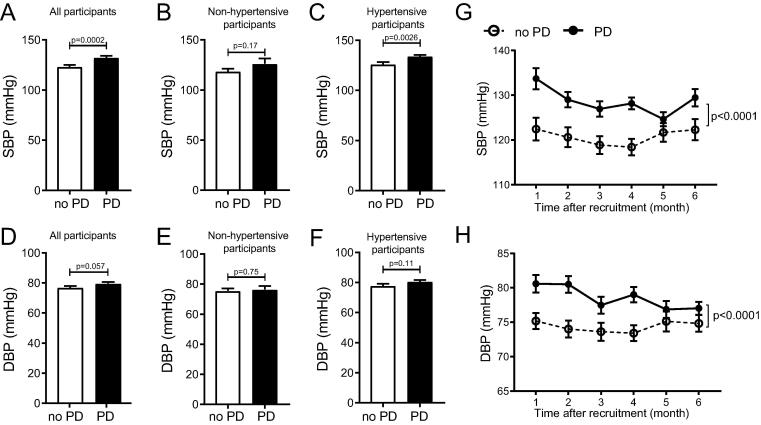
**Participants with PD have higher blood pressure. A-C**, SBP of all participants (A), non-hypertensive participants (B), and hypertensive participants (C) with or without PD. **D-F**, DBP of all participants (D), non-hypertensive participants (E), and hypertensive participants (F) with or without PD. **G-H**, SBP and DBP of individuals with or without PD in the follow-up study. n = 59:75 for A and D, 23:16 for B and E, 36:59 for C and F, and 34:44 for G and H. Student’s *t*-test was used for statistical analysis in A-F and Two-way ANOVA followed by Sidak’s multiple comparison test in G and H.

To study the impacts of PD and oral/gut microbiota on BP, we recruited 134 participants, 39 of which were without HTN (no HTN) and 95 had HTN (Table 1). We first analyzed the association between PD and HTN at baseline. When the whole cohort was analyzed together, participants with PD manifested significantly higher systolic BP (SBP) than those without PD (no PD) (Fig. 1A). When non-hypertensive and hypertensive participants were analyzed separately, non-hypertensive participants with PD showed a strong trend to have higher SBP than no PD (Fig. 1B), and hypertensive participants with PD showed significantly higher SBP than no PD (Fig. 1C). Participants with PD were also inclined to have higher diastolic BP (DBP) than no PD either among the whole cohort or being divided into non-hypertensive and hypertensive groups, although no statistical difference was detected (Fig. 1D-F). These results are largely consistent with previous reports[18], [19].

**Table 1:** Demographics of enrolled participants.

Characteristics	no HTN		HTN		P values (no HTN VS HTN)
	no PD (n = 23)	PD (n = 16)	no PD (n = 36)	PD (n = 59)	
Sex	16 women; 7 men	11 women; 5 men	27 women; 9 men	32 women; 27 men	0.435
Age (years)	62.87 ± 2.03	67.38 ± 1.56	67.42 ± 1.82	68.14 ± 0.79	0.057
Height (cm)	163.7 ± 1.25	163.6 ± 1.90	160.6 ± 1.58	163.8 ± 1.07	0.430
Weight (kg)	61.13 ± 1.89	65.46 ± 2.29	62.53 ± 2.04	66.14 ± 1.50	0.332
BMI (kg/m^2^)	22.81 ± 0.69	24.51 ± 0.92	24.12 ± 0.57	24.6 ± 0.46	0.175
Systolic blood pressure, (mmHg)	118.6 ± 2.64	126.2 ± 5.4	126.1 ± 2.25	134.6 ± 1.46	0.0007
Diastolic blood pressure, (mmHg)	75.55 ± 1.56	76.44 ± 2.4	77.82 ± 1.32	80.6 ± 1.1	0.022
Probing depth (mm)	<4	≥4	<4	≥4	NA
Attachment loss (mm)	<3	≥3	<3	≥3	NA
Drinkers, n (%)	2 (8.70%)	1 (6.25%)	3 (8.33%)	5 (8.47%)	0.889
Diabetes mellitus, n (%)	0 (0.00%)	0 (0.00%)	4 (11.12%)	10 (16.95%)	NA
Follow-up participants, n	15 (9 women; 6 men)	11 (8 women; 3 men)	19 (13 women; 6 men)	33 (16 women; 17 men)	0.203
Antihypertensive treatment, n (%)	0 (0.00%)	0 (0.00%)	30 (83.34%)	48 (81.36%)	NA
Naïve to antihypertensive treatment, n (%)	NA	NA	6 (16.66%)	11 (8.64%)	NA

Data are shown as mean ± SD or n (%). PD, periodontitis. HTN, hypertension. BMI, Body Mass Index. NA, not applicable.

Pearson's chi-square test was used for statistical analyses of sex, drinkers, and follow-up participants between no HTN and HTN.

Student’s *t*-test was used for statistical analyses of age, height, weight, BMI, and blood pressure between no HTN and HTN.

In addition to these cross-sectional analyses at baseline, we conducted a 6-month follow-up study. Both SBP and DBP were substantially higher in participants with PD than no PD throughout the follow-up (Fig. 1G**, H**). Together these results demonstrated that participants with PD had persistently higher BP than no PD.

### Altered diversity of oral and gut microbiota in participants with HTN

**Fig. 2:**
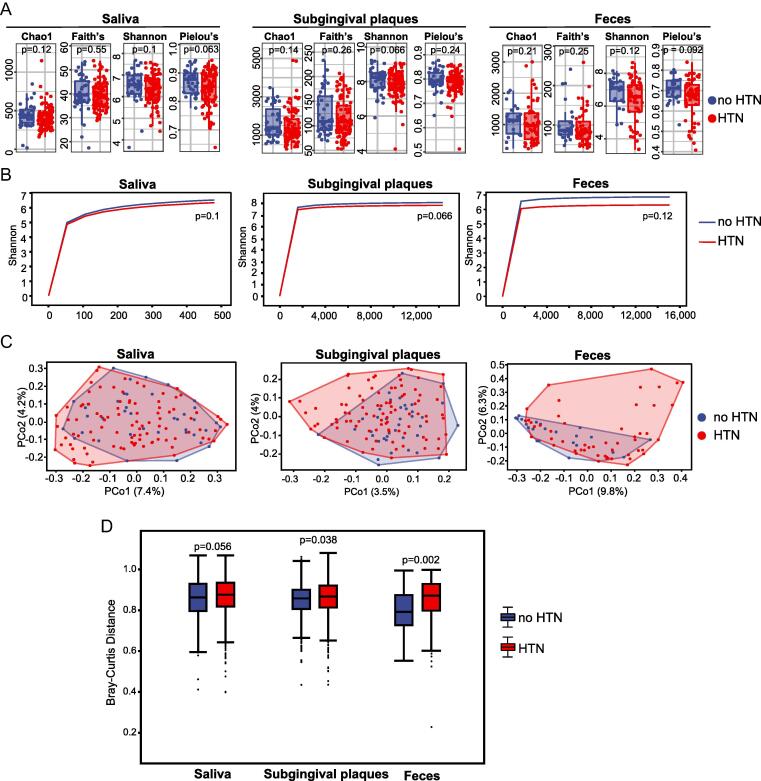
**Shifts of microbiota diversity in oral cavity and intestine of participants with HTN. A**, Chao1, Faith’s phylogenetic diversity (Faith’s), Shannon index, and Pielou’s evenness (Pielou’s) of oral (saliva and subgingival plaques) microbiota and gut (feces) microbiota in participants with or without HTN. **B**, Rarefaction curves of oral and gut microbiota in participants with or without HTN. **C**, PCoA of oral and gut microbiota in participants with or without HTN. **D**, Bray-Curtis distance of oral and gut microbiota in participants with or without HTN. All microbiota was analyzed using 16S rRNA gene sequencing. n = 39:94 for saliva, 39:93 for subgingival plaques, and 24:52 for feces. Kruskal-Wallis rank sum test was used for statistical analysis in A and permutational multivariate analysis of variance in D.

We identified 368 genera under 38 phyla in saliva, 265 genera under 31 phyla in subgingival plaques, and 113 genera under 28 phyla in feces of our study population using 16S rRNA gene sequencing (Table S1). Analyses of alpha (α)-diversity demonstrated that microbiota of HTN participants tended to have lower Chao1 richness, Faith’s phylogenetic diversity, Shannon diversity index, and Pielou’s Evenness index in all 3 sample types compared to no HTN participants, although none of the differences reached statistical significance (Fig. 2A). Shannon rarefaction curves also supported the trend of decrease in α-diversity in the HTN group, especially for gut microbiota (Fig. 2B), consistent with the results previously reported[20]. PCoA was performed to assess beta (β)-diversity of the microbiota (Fig. 2C). Permutational multivariate analysis of variance based on Bray-Curtis distance demonstrated statistically significant differences in β-diversity of subgingival microbiota and gut microbiota between no HTN and HTN group (Fig. 2D). The difference in β-diversity of salivary microbiota also showed a statistical trend (p = 0.056) between the two groups (Fig. 2D). Results of Adonis test based on Bray-Curtis did not show significant impact of diabetes or antihypertensive treatment on microbiota (Table S2).

### The composition of oral and gut microbiota shifts in participants with HTN

**Fig. 3:**
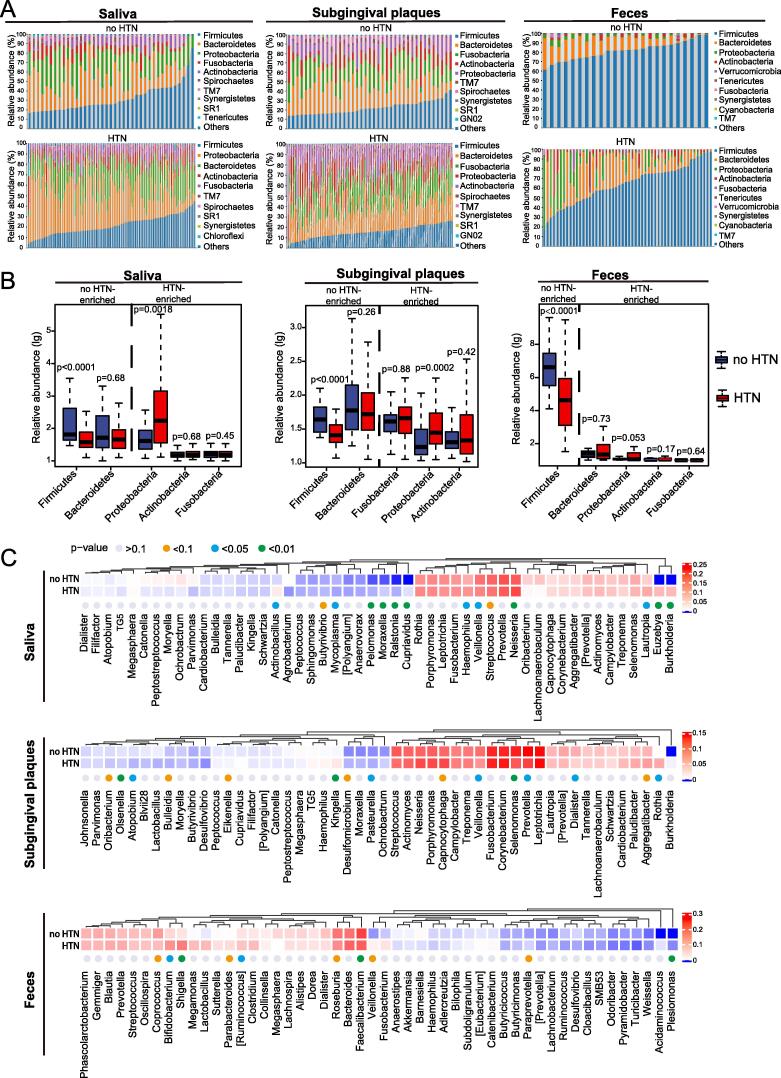
**Compositional alterations of oral and gut microbiota in participants with HTN. A**. Stacked bar plots showing relative abundances of oral (saliva and subgingival plaques) microbiota and gut (feces) microbiota at phylum level in participants with or without HTN. **B**. Box plots showing differential enrichment of the top 5 phyla in participants with or without HTN. **C**. Clustering heatmaps showing relative abundances of the top 50 genera in participants with or without HTN. All microbiota was analyzed using 16S rRNA gene sequencing. n = 39:94 for saliva, 39:93 for subgingival plaques, and 24:52 for feces. Mann-Whitney U rank sum (MW) test was used for statistical analysis in B and Kruskal-Wallis rank sum test in C.

Stacked bar plots of relative abundances at the phylum level demonstrated evident differences in oral and gut microbiota between no HTN and HTN (Fig. 3A). *Firmicutes, Bacteroidetes, Proteobacteria, Actinobacteria,* and *Fusobacteria* were predominant phyla in saliva and subgingival plaques, and *Firmicutes* and *Bacteroidetes* were predominant phyla in feces of both no HTN and HTN (Fig. S1**,** relative abundance > 10%). *Firmicutes* significantly decreased in HTN compared to no HTN in all 3 types of samples and *Proteobacteria* substantially increased in saliva and subgingival plaques of HTN (Fig. 3B). Moreover, in saliva and subgingival plaques, *Bacteroidetes* were enriched in no HTN, whereas *Actinobacteria* and *Fusobacteria* were enriched in HTN group; in feces, *Bacteroidetes*, *Proteobacteria*, *Actinobacteria* and *Fusobacteria* were enriched in HTN group, although these differences did not reach statistical significance (Fig. 3B). We further analyzed the composition of microbiota at the genus level. Among the top 50 genera, there were 14 genera in salivary microbiota, 15 genera in subgingival microbiota, and 10 genera in gut microbiota that showed substantial differences between no HTN and HTN (p < 0.1) (Fig. 3C).

### Associations between oral/gut microbiota and BP and other clinical parameters

**Fig. 4:**
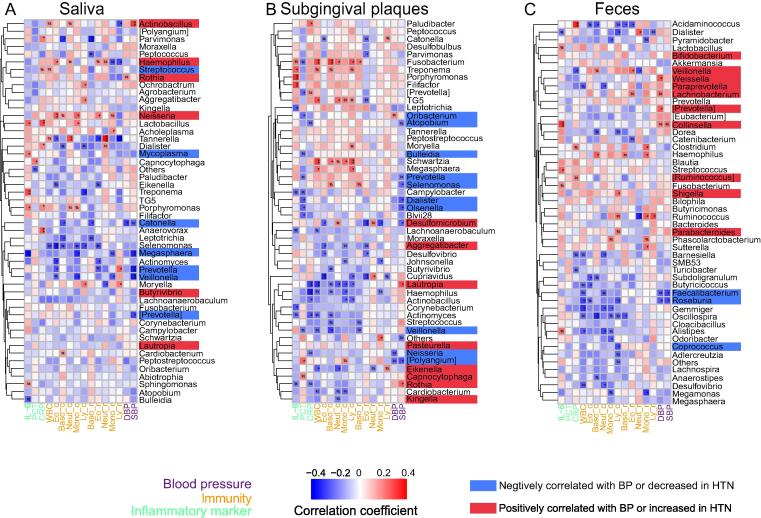
**Associations between oral/gut microbiota and blood pressure and other clinical parameters.** Heatmaps of Spearman’s correlation coefficients between clinical parameters (blood pressure, immunity, inflammatory markers) and relative abundances of the top 50 genera in microbiota of saliva (**A**), subgingival plaques (**B**), and feces (**C**). IL-6: Interleukin-6, PCT: Procalcitonin, CRP: C-reactive protein, WBC: White blood cells, Eo_c: Eosinophil count, Baso_c: Basophil count, Neut_c: Neutrophil count, Mono_c: Monocyte count, Ly_c: Lymphocyte count, Eo_r: Eosinophil ratio, Baso_ r: Basophil ratio, Neut_ r: Neutrophil ratio, Ly_ r: Lymphocyte ratio, Mono_r: Monocyte ratio, DBP: Diastolic blood pressure, SBP: Systolic blood pressure. n = 77 for saliva and subgingival plaques, and 76 for feces. #p(FDR) < 0.1, *p(FDR) < 0.05, **p(FDR) < 0.01, ***p(FDR) < 0.001.

To evaluate the clinical significance of the alterations of oral and gut microbiota in HTN, Spearman’s correlation analyses were performed to assess the associations between clinical parameters and the relative abundances of the top 50 genera in saliva, subgingival plaques, and feces. We first investigated the associations between microbiota and BP. SBP had a significant positive correlation with *Actinobacillus* and negative correlations with *Catonella, Megasphaera, Prevotella, Veillonella,* and *[Prevotella]* in saliva (Fig. 4A); significant positive correlations with *Lautropia* and *Rothia*, and negative correlations with *Atopobium, Prevotella, Selenomonas, Dialister, Olsenella, Desulfomicrobium,* and *[Polyangium]* in subgingival plaques (Fig. 4B). DBP had a significant positive correlation with *Rothia* and a negative correlation with *Catonella* in saliva (Fig. 4A); significant positive correlation with *Oribacterium*, and negative correlations with *[Polyangium]* and *Neisseria* in subgingival plaques (Fig. 4B). As for gut microbiota, both SBP and DBP had a significant positive correlation with *Collinsella*, and negative correlations with *Faecalibacterium* and *Roseburia* (Fig. 4C); DBP had significant positive correlations with *Weissella*, *Lachnobacterium,* and *[Prevotella]* (Fig. 4C). All genera with significant positive correlations with BP or those significantly increased in HTN (based on Fig. 3**C**) were highlighted in red, and all genera with significant negative correlations with BP or those significantly decreased in HTN (based on Fig. 3**C**) were highlighted in blue (Fig. 4A-C).

Given the importance of inflammation in HTN[21], we then analyzed the associations between inflammatory markers and microbiota. Interleukin-6 (IL-6) had significant correlations with 7 salivary, 10 subgingival, and 6 fecal genera (Fig. 4A-C). Procalcitonin had significant correlations with 2 salivary and 12 subgingival genera (Fig. 4 **A-B**). C-reactive protein (CRP) had significant correlation with 6 salivary, 9 subgingival, and 6 fecal genera (Fig. 4A-C). Notably, PD-associated genera such as salivary *Porphyromonas*, subgingival *Porphyromonas*, *Fusobacterium* and *Treponema* had significant positive correlations with plasma IL-6, and salivary *Porphyromonas* had significant positive correlation with plasma CRP (Fig. 4A-B).

We further analyzed the associations between immunological parameters and microbiota, focusing on the red- or blue-highlighted microbes. Leukocyte counts and leukocyte ratios manifested similar pattern of correlations with abundances of oral/gut microbiota (Fig. 4A-C). Interestingly, *Veillonella* in each sample type significantly correlated with at least one leukocyte count/ratio (Fig. 4A-C). We also identified correlations between oral/gut microbiota and other clinical parameters, including anthropometric measurements, immunoglobulins, red blood cells, platelets, liver and kidney function, metabolic traits, and electrolytes (Fig. S2).

Taken together, these results revealed close correlations between oral/gut microbiota and BP/other clinical parameters, and suggested that microbiota could directly affect BP or influence BP by modulating blood pressure-related clinical parameters.

### Communications between oral and gut microbiota in HTN

The importance of oral-gut microbial transmission has been recognized in different diseases[14], [22]. We next extended our analysis to investigate the potential communications between oral and gut microbiota in HTN. Microbial abundance co-correlation networks illustrated that the HTN-enriched networks had more interconnections than no HTN-enriched ones in all 3 sample types (Fig. S3A).

**Fig. 5:**
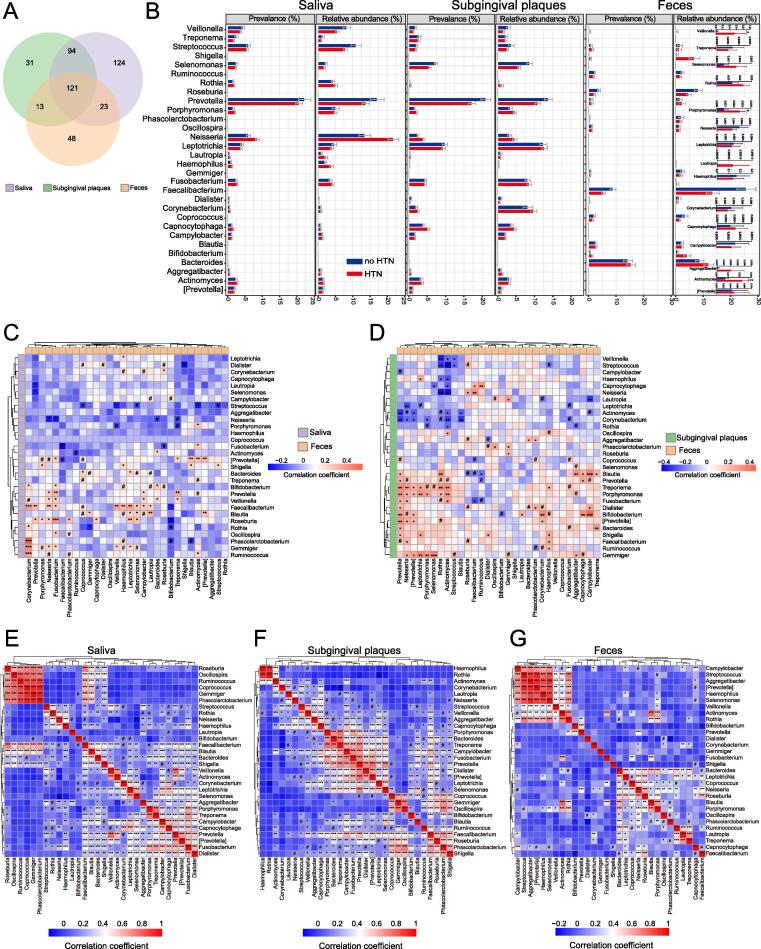
**Communications between oral and gut microbiota in participants with or without HTN. A**. Venn diagrams showing the unique and shared OTUs among saliva, subgingival plaques, and feces. The 30 most abundant genera out of the 121 shared genera among salivary, subgingival, and gut microbiota were used for analyses in B-G. **B**. Prevalence and relative abundances of the top 30 shared genera in no HTN and HTN. The insets of the ‘Feces’ column show the relative abundance of the 15 low-abundance gut genera on enlarged y axes. **C-D**. Heatmaps of Spearman’s correlation coefficients between relative abundances of shared genera in salivary (C) or subgingival (D) microbiota and those in gut microbiota. **E-G**. Heatmaps of Spearman’s correlation coefficients among relative abundances of the top 30 shared genera within salivary (E), subgingival (F), or gut (G) microbiota. n = 133 for saliva, 132 for subgingival plaques, and 76 for feces in A and E-G. n = 39:94 for saliva, 39:93 for subgingival plaques, and 24:52 for feces in B. n = 75 for saliva, subgingival plaques, and feces in C and D. #p(FDR) < 0.1, *p(FDR) < 0.05, **p(FDR) < 0.01, ***p(FDR) < 0.001.

Comparison of the microbiota among different types of samples using the Venn diagram identified 121 shared genera that coexisted in saliva, subgingival plaques, and feces (Fig. 5A). The relative abundance and prevalence of the top 30 shared genera showed reciprocal relationships between oral samples and gut samples (high in oral samples and low in gut samples, vice versa) (Fig. 5B). Genera such as *Veillonella*, *Porphyromonas*, *Streptococcus* and *Prevotella* that are usually found in the oral cavity were more prevalent or abundant in the gut of HTN than no HTN participants (Fig. 5B). The most pronounced change was the sharp increase of the relative abundance of *Veillonella* in the gut of HTN versus no HTN participants (Fig. 5B). These data together suggested that orally-originated genera such as *Veillonella* ectopically colonized in the gut and that HTN promoted such ectopic colonization.

We then analyzed the correlations of the top 30 shared genera between oral and gut sample types and paid special attention to *Veillonella*. The relative abundance of gut *Veillonella* was significantly correlated with salivary *Gemmiger*, *Faecalibacterium*, *Porphyromonas*, and *Streptococcus* (Fig. 5C). However, there was no significant correlation between gut *Veillonella* and subgingival microbiota (Fig. 5D). These results suggested that the potential ectopic colonization of *Veillonella* more likely involved the salivary rather than subgingival genus. SourceTracker analysis showed that HTN tended to increase the saliva-originated proportion while decrease the subgingival plaques-originated proportion in gut microbiota (Fig. S3B), indicating that the saliva-gut communication of *Veillonella* was associated with HTN. We further inspected the relationship between *Veillonella* and the above-mentioned 4 genera (*Gemmiger*, *Faecalibacterium*, *Porphyromonas*, and *Streptococcus*) within each sample type (Fig. 5E**-G**). The relationship between *Streptococcus* and *Veillonella* caught our close attention because only the correlations between these two genera within each sample type (saliva, subgingival plaques, and feces) were significant and positive (Fig. 5E**-G**). Previous reports have considered *Streptococcus* and *Veillonella* common colonizers in the gastrointestinal tract[23], [24], and *Streptococcus* is able to promote the growth of *Veillonella* by producing lactic acid[25]. It was plausible that salivary *Streptococcus* and *Veillonella* co-colonized ectopically in the gut to affect BP. Therefore, these data suggested potential communications between oral and gut microbiota in HTN.

Analyses of the correlations of the top 30 shared genera between saliva and subgingival plaques unveiled 183 significant correlations, most strikingly among which the significant positive correlations between 10 genera in saliva and 9 genera in subgingival plaques clustered together, suggesting strong communications of microbiota between saliva and subgingival plaques as expected (Fig. S3C).

### Oral-gut transmission of microbiota in HTN at species level

To further investigate the communications between oral and gut microbiota, we performed shotgun metagenomic sequencing of oral and gut samples from 24 no HTN participants and 36 HTN participants. Co-correlation networks of predominant species illustrated that the HTN-enriched networks had more interconnections than no HTN-enriched ones in all 3 sample types (Fig. S4). Linear discriminant analysis effect size (LEfSe) revealed differentially enriched microbial species between HTN and no HTN (Fig. S5A-C). Interestingly, PD-associated species (*Porphyromonas gingivalis*, *Tannerella forsythia*, *Treponema denticola*, and *Porphyromonas endodontalis*) were significantly enriched in subgingival plaques of HTN participants (Fig. S5B). LEfSe also revealed differentially enriched microbial functional modules between HTN and no HTN (Fig. S6A, B). The majority of the microbial gene functions in oral samples overlapped with those in gut samples, indicating shared functional modules between oral and gut microbiota (Fig. S6C). Multiple microbial functional modules significantly correlated with BP and other clinic parameters (Fig. S7).

**Fig. 6:**
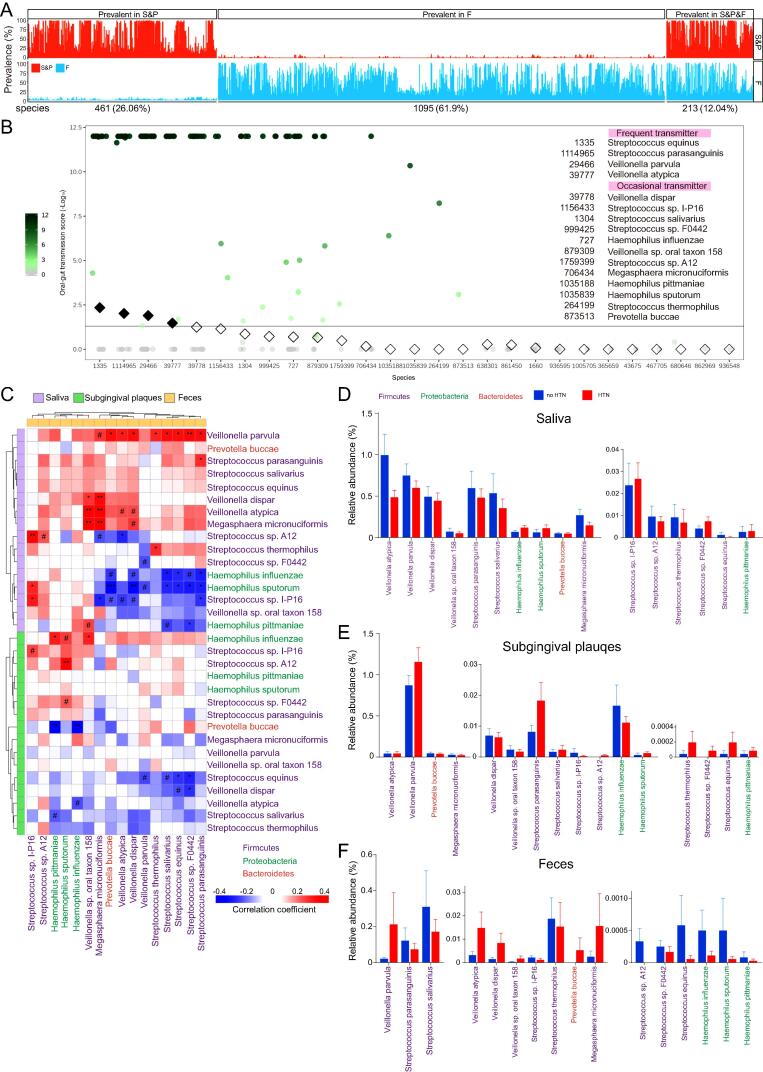
**Oral-gut microbiota transmission in participants with or without HTN at species level.** All microbiota was analyzed using metagenomic sequencing. **A**. Overview of microbial species prevalent in oral samples (saliva & subgingival plaques, abbreviated as S&P), gut samples (feces, abbreviated as F), or both oral and gut samples (S&P&F). **B**. Oral-gut transmission scores of shared species in oral and gut microbiota based on metaSNV analysis. Filled diamonds indicate p < 0.05 by Wilcoxon rank-sum test and the corresponding species are classified as frequent transmitters. Open diamonds indicate p > 0.05 by Wilcoxon rank-sum test but p < 0.05 by Mann-Whitney U rank sum test, and the corresponding species are classified as occasional transmitters. **C**. Heatmap of Spearman’s correlation coefficients between relative abundances of the 16 oral-gut transmitters in oral (salivary and subgingival) microbiota and those in gut microbiota. **D-F**. Relative abundances of frequent transmission and occasional transmission species in saliva (D), subgingival plaques (E), and feces (F). n = 60 in A-C, and 24:36 in D-F. #p(FDR) < 0.1, *p(FDR) < 0.05, **p(FDR) < 0.01, ***p(FDR) < 0.001.

We detected 461 species prevalent in oral samples (saliva and subgingival plaques combined), 1095 species prevalent in feces, and 213 prevalent in both oral and gut samples (Fig. 6A). Previous reports have suggested that tracking microbial communities at the level of strains rather than species is more reliable for establishing and quantifying microbiota transmission[26], [27]. We therefore analyzed the SNVs in our metagenomic sequencing data. Based on Manhattan distance obtained from the SNV distance matrix of oral-gut sample pairs, 16 out of the 213 species shared in oral and gut samples were identified as oral-gut transmitters, 4 of which (*Streptococcus equinus*, *Streptococcus parasanguinis*, *Veillonella parvula*, and *Veillonella atypica*) were categorized as frequent transmitters and the other 12 as occasional transmitters according to their oral-gut transmission scores (Fig. 6B). These 16 oral-gut transmitters belonged to 5 genera (*Veillonella*, *Streptococcus*, *Haemophilus*, *Prevotella*, and *Megasphaera*) under 3 phyla (*Firmcutes*, *Proteobacteria*, and *Bacteroidetes*) and most of them were more abundant in oral samples (especially saliva) than feces (Fig. 6C-F). Similar to the negative correlation between *Streptococcus* and *Veillonella* at the genus level (Fig. 5C-D), most of the associations between oral *Streptococcus* spp*.* and gut *Veillonella* spp*.* were negative correlations (Fig. 6C).

The impacts of HTN on the relative abundances of these 16 species in feces were largely consistent with those at the genus level, except for *Megasphaera micronuciformis* (and its belonging genus *Megasphaera*) (Fig. 6F, S8). Importantly, all 4 species under the genus of *Veillonella* (*Veillonella parvula*, *Veillonella atypica*, *Veillonella dispar*, and *Veillonella* sp. *oral taxon 158*) were increased in HTN compared to no HTN group (Fig. 6F). Therefore, the 16 oral-gut transmitting species, particularly *Veillonella* spp, may play important roles in HTN.

### Sustained oral-gut transmission of microbiota in HTN

**Fig. 7:**
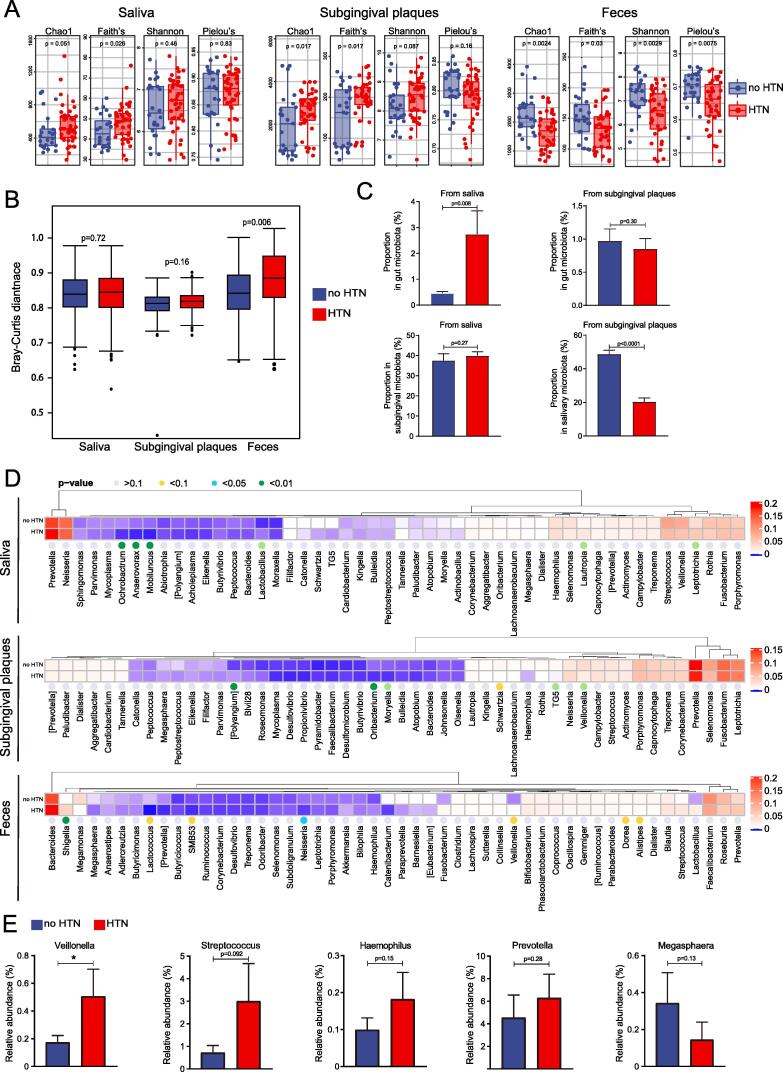
**Sustained oral-gut transmission in patients with HTN after 6 months. A**. Chao1, Faith’s phylogenetic diversity (Faith’s), Shannon index, and Pielou’s evenness (Pielou’s) of oral (saliva and subgingival plaque) microbiota and gut microbiota in participants with or without HTN. **B**. Bray-Curtis distance of oral and fecal microbiota in participants with or without HTN. **C**. SourceTracker analysis to estimate microbial communications from oral cavity to gut and within oral cavity. **D**. Clustering heatmaps showing relative abundances of the top 50 genera in participants with or without HTN. **E**. Relative abundances of the 5 oral-gut-transmitting genera in feces. All microbiota was analyzed using 16S rRNA gene sequencing. n = 24:43 for saliva, 25:43 for subgingival plaques, and 24:38 for feces. Kruskal-Wallis rank sum test was used for statistical analysis in A and B, Student’s *t* test in C and E, and permutational multivariate analysis of variance in D. *p < 0.05.

Both oral and gut microbiota fluctuate over time[28], [29]. We conducted a follow-up study in 52 HTN participants and 26 controls to address whether the alterations of microbiota in HTN and the oral-gut transmission sustained. Different from the lower α-diversity between HTN and no HTN group 6 months ago, 16S rRNA gene sequencing revealed that both salivary and subgingival microbiota of HTN subjects had higher Chao1 richness, Faith’s phylogenetic diversity, and Shannon diversity index (Fig. 7A). These 3 indexes in gut microbiota and Pielou’s Evenness index for all sample types maintained the similar trend as before (Fig. 7A). PCoA and permutational multivariate analysis of variance based on Bray-Curtis distance demonstrated a significant difference in β-diversity of gut but not oral microbiota between no HTN and HTN group (Fig. 7B, S9). The difference in diversity between these two time points suggested that microbiota in the gut had less fluctuation than in the oral cavity.

SourceTracker analysis in the follow-up cohort suggested an increase of oral-gut communication in HTN, particularly manifested by the significantly increased proportion of saliva-originated microbes in gut microbiota (Fig. 7C). The composition of the top 50 genera in each sample type was similar to that 6 months ago, although the significantly different genera between no HTN and HTN varied (Fig. 7D). Among the 5 genera that the 16 oral-gut transmitting species belong to, the relative abundance of *Veillonella* significantly increased in gut microbiota of HTN participants (Fig. 7E), consistent with the results 6 months ago (Fig. S8). Together these data indicated the stability of the oral-gut transmission and ectopic colonization of oral microbiota in HTN.

### A causal relationship between oral-gut microbial transmission and HTN

**Fig. 8:**
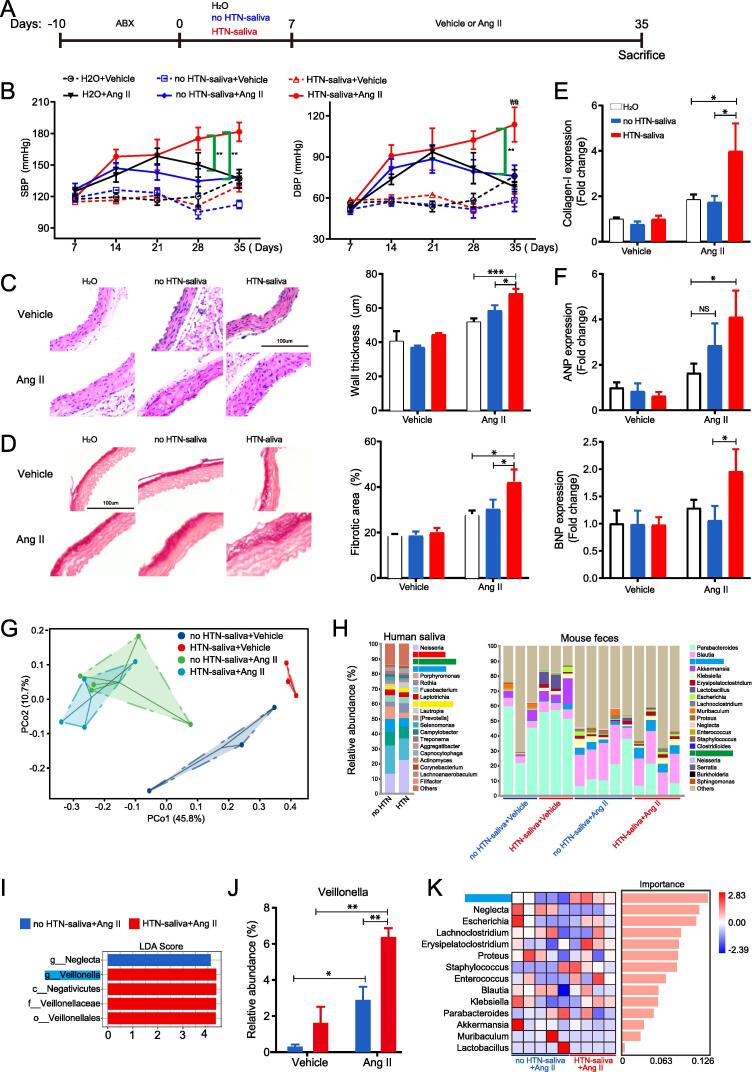
**Ectopic colonization of salivary microbiota from HTN participants in mouse intestine aggravates HTN. A**. Schematic illustration of experimental design. ABX, antibiotic cocktail; Ang II, Angiotensin II. **B**. Noninvasive tail-cuff monitoring of SBP and DBP in mice. **C**. Representative H&E staining of thoracic aortas (left) and the quantification of wall thickness (right). Magnification = 200 ×. Three fields were randomly chosen in each sample for quantification. **D**. Representative Picrosirius red staining of thoracic aortas (left) and the quantification of fibrotic areas (right). Magnification = 200 ×. Three fields were randomly chosen in each sample for quantification. **E-F**. QRT-PCR analysis of collagen-I in thoracic aortas (E) as well as atrial natriuretic peptide (ANP) and B-type natriuretic peptide (BNP) in left ventricles (F). **G**. PCoA of mouse feces. **H**. Stacked bar plots showing relative abundances of the top 20 microbial genera in human saliva and mouse feces. **I**. LEfSe of mouse gut microbiota. The threshold of LDA score was 2. **J**. The relative abundance of Veillonella in mouse gut microbiota. **K**. Random forest regression analysis of mouse gut microbiota at genus level. The heatmap represents relative abundances of microbes. n = 3:3:3 for H2O + Vehicle vs no HTN-saliva + Vehicle vs HTN-saliva + Vehicle; n = 5:5:4 for H2O + Ang II vs no HTN-saliva + Ang II vs HTN-saliva + Ang II. ## p < 0.01 for no HTN-saliva + Ang II vs HTN-saliva + Ang II at day 35 in (B); *p < 0.05, **p < 0.01, *** p < 0.001.

To investigate the potential causal link between oral-gut transmitters and HTN, we performed saliva microbiota transplantation experiments in ABX-pretreated recipient mice infused with vehicle (saline) or Ang II infusion (Fig. 8A). Tail-cuff BP measurements revealed that mice received saliva from HTN participants (HTN-saliva) had significantly higher SBP and/or DBP than those received saliva from no HTN participants (no HTN-saliva) or water after Ang II infusion (Fig. 8B). Ang II-induced hypertrophy and fibrosis of aortas were significantly increased in mice received HTN-saliva compared to those received no HTN-saliva or water (Fig. 8C-D). Moreover, Ang II-induced expression of collagen-I in aortas and ANP and BNP in hearts was markedly higher in mice received HTN-saliva (Fig. 8E-F). Mesenteric arteries isolated from mice received HTN-saliva manifested stronger contraction in response to phenylephrine and Ang II (Fig. S10). These results demonstrated that the ectopic colonization of HTN-saliva in mouse intestine exacerbated angiotensin II-induced HTN.

To validate the importance of the oral-gut transmitters identified in our clinical study, we performed 16S rRNA gene sequencing on human saliva and feces of mice received human saliva. PCoA demonstrated that the gut microbial community composition between mice received no HTN-saliva and those received HTN-saliva were completely separated but more similar after Ang II infusion (Fig. 8G). ABX pretreatment depleted all oral-gut transmitting genera (*Veillonella*, *Streptococcus*, *Prevotella*, *Haemophilus*, and *Megasphaera*) in mouse gut microbiota (Fig. S11). Although the human saliva samples contained all transmitting genera (4 genera in Fig. 8H, *Megasphaera* was not shown due to low abundance), only *Veillonella* successfully colonized and presented in the intestine of all saliva-treated mice (*Streptococcus* was only detected in two mice, Fig. 8H). Importantly, *Veillonella* was more enriched (Fig. 8I) and more abundant in the gut microbiota of mice received HTN-saliva and infused with Ang II (Fig. 8J). Furthermore, the importance of *Veillonella* ranked the highest according to random forest regression analysis of gut microbiota of mice received human saliva and infused with Ang II (Fig. 8K).

Taken together, the results of these animal experiments are largely consistent with those of our clinical study. Mice transplanted with HTN-saliva had significantly elevated BP. A higher abundance of saliva-derived *Veillonella* in mouse gut may be a causal link between oral-gut microbial transmission and HTN.

## Discussion

PD is an oral microbiota-related disease and has been linked to HTN[19], [30], [31]. However, few studies have focused on direct relationships between oral microbiota and HTN or oral-gut microbiota transmission in HTN. Herein, we for the first time reported a comprehensive analysis of oral and gut microbiota between no HTN and HTN by 16S rRNA gene sequencing and metagenomic sequencing. Besides substantiating the association between PD and HTN, our data revealed the differences in diversity and composition of oral and gut microbiota between no HTN and HTN, as well as established correlations between oral/gut microbiota and clinical parameters. We identified oral-gut microbial transmitters at both genus and species levels. Most notably, *Veillonella* spp., among 16 oral-gut transmitting species, may exert crucial functions in HTN.

Our results have strengthened the association between PD and HTN. According to a recent systematic review and *meta*-analysis, patients with PD exhibit 4.5 and 2.0 mmHg higher SBP and DBP respectively than those without PD[32]. Our results confirmed such association between PD and BP in both hypertensive and normotensive participants. The differences in BP between no PD and PD in this study were greater than those reported before[19], likely because of the difference in study populations. More importantly, our data for the first time demonstrated that participants with PD had higher BP not only in cross-sectional but also in follow-up studies, suggesting that PD might affect both onset and progression of HTN. We also observed a trend of decrease in BP for both PD and no PD participants during the follow-up, likely because of change in seasons (from winter to summer in this case) and/or ease of anxiety of the participants over time[33], [34]. Our study and previous studies together strongly support that PD is an important risk factor for HTN and needs to be monitored continuously.

We have provided comprehensive evidence to support strong associations between oral microbiota and HTN. We observed a host of oral genera that were significantly altered in HTN and/or significantly correlated with BP. Among these genera, the relative abundances of oral *Streptococcus* and *Prevotella* were significantly decreased in HTN participants, consistent with the results of a previous study using subgingival plaques from older women[8]. It has been reported that *Veillonella* is an important nitrate-reducing genus in the oral cavity linked to lower BP[35]. Interestingly, dietary nitrates have beneficial blood pressure-lowering effects, and oral microbiome plays an important role in that the commensal bacteria are required to convert nitrate to nitrite, the latter of which may be further converted to nitric oxide to lower blood pressure[36], [37]. Consistently, the relative abundance of oral *Veillonella* was markedly decreased in HTN and negatively correlated with BP in our study, although other nitrate-reducers such as *Neisseria*, *Haemophilus* and *Rothia* were increased in HTN. The relative abundances of oral *Burkholderia*, *Lautropia*, and *Ralstonia* were significantly increased in HTN participants of our study. Interestingly, these 3 genera have been shown to correlate with pulmonary arterial HTN[38], [39]. Moreover, we established new associations between HTN and oral microbes, including *Actinobacillus*, *Aggregatibacter*, *Atopobium*, *Bulleidia*, *Cupriavidus*, *Desulfomicrobium*, *Eikenella*, *Euzebya*, *Kingella*, *Moraxella*, *Olsenella*, *Pasteurella*, *Pelomonas*, and *Selenomonas*. Our results also revealed that PD-associated pathogens such as *Porphyromonas*, *Fusobacterium* and *Treponema* were more abundant in subgingival plaques of HTN participants, and positively correlated with IL-6 and/or CRP. These 3 PD-associated pathogens and *Prevotella* have been reported to play a pro-inflammatory role, which may be mediated by the microbial toxins such as gingipain derived from *Porphyromonas gingivalis* and lipopolysaccharide derived from *Prevotella intermedia*[40], [41], [42], [43]. These associations further substantiated the importance of oral microbiota in HTN.

The most novel finding of this study is the identification of important oral-gut transmitting microbes in HTN. Our overall findings of gut microbiota in HTN participants were consistent with those previously reported (e.g., similar α-diversity and genus enrichment)[20]. We further explored the influence of oral-gut transmitting microbes and discovered oral-gut microbial transmitters that might participate in BP regulation. SourceTracker analyses in both cross-sectional and follow-up cohorts suggested increases of transmission between oral and gut microbiota in HTN participants. Correlation analyses at genus level indicated communications between oral and gut microbiota, and orally-originated genera such as *Veillonella* and *Streptococcus* might co-colonize ectopically in the gut to affect BP. Further analysis at species level identified 16 oral-gut transmitting species under 5 genera (*Veillonella*, *Streptococcus*, *Haemophilus*, *Prevotella*, and *Megasphaera*) that may play important roles in HTN. It is worth to point out that all these 5 genera are the core oral taxa with high relative abundances[44]. Intriguingly, except for and *Megasphaera*, the rest 4 genera fall into the oral-gut microbial transmission category as reported before[9]. Given the pivotal role of gut microbiome on sympathetic nerves, oral-derived gut bacteria could also influence BP via the brain-gut axis after colonization[45].

*Veillonella* has earned an eminent place among the oral-gut microbial transmitters in HTN. Relative abundances of fecal *Veillonella* significantly increased in HTN participants of both cross-sectional and follow-up cohorts. Among the 16 oral-gut transmitting species, all 4 *Veillonella* species were enriched in HTN participants. Ectopic colonization of oral microbiota in the intestine is usually considered a hallmark of diseases[12], [46], [47]. Orally-derived gut microbes including *Veillonella* have been used to establish a diagnostic model to distinguish and predict colorectal cancer with a high efficacy[48]. *Veillonella* is also a potential pathobiont expanded in autoimmune hepatitis and associated with disease status[49]. In addition, oral microbiota such as *Veillonella atypical* and *Veillonella dispar* may translocate to the gut of patients with schizophrenia[50]. Our results confirmed that *Veillonella* was a strong oral-gut transmitter. Particularly, the colonization of saliva-derived *Veillonella* in the gut of mice transplanted with human saliva suggested that the oral cavity was a reservoir for the ectopic colonization of *Veillonella*. Saliva-derived *Veillonella* was more enriched in Ang II-infused mice, indicating that it was more likely to colonize under HTN status. More importantly, higher abundance of *Veillonella* colonization was accompanied by significantly higher BP in mice transplanted with saliva from HTN participants, suggesting that the ectopic colonization of oral *Veillonella* in the gut was an important factor influencing the development and progression of HTN. Our study used male mice only, which was a limitation given the sex differences in hypertensive animal models[51].

Taken all together, this study has reinforced the association between PD and HTN, established strong correlations between oral and gut microbiota, between oral/gut microbiota and HTN/HTN-associated clinical parameters, as well as identified HTN-related oral-gut transmitting microbes. These data have comprehensively demonstrated the roles of PD and oral microbiota in HTN, particularly revealing the importance of oral-gut transmission of microbes such as *Veillonella* spp. These findings support joint control of PD and HTN, and the identification of orally-derived microbes in the gut may provide novel strategies for prevention, diagnosis and therapy of HTN.

## Conclusions

PD is constantly associated with HTN. Dysbiosis of oral and gut microbiota highlights the association between PD and HTN. Oral-gut transmission of microbes, particularly *Veillonella* spp., is an important mechanism contributing to HTN. Regular monitoring PD and targeting oral-gut microbial transmission may become effective strategies to improve the prevention and treatment of HTN.

## Compliance with ethics requirement

The study protocol was approved by the Institutional Review and Ethics Board of Shanghai Ninth People’s Hospital, Shanghai Jiao Tong University School of Medicine (SH9H-2018-T66-3). Informed consent was signed by all subjects before enrollment.

## CRediT authorship contribution statement

**Bo-Yan Chen:** Conceptualization, Software, Methodology, Formal analysis, Writing – review & editing, Investigation, Validation, Data curation, Project administration, Visualization. **Wen-Zhen Lin:** Investigation, Validation, Data curation, Project administration. **Yu-Lin Li:** Investigation, Validation, Data curation, Project administration. **Chao Bi:** Investigation. **Lin-Juan Du:** Investigation. **Yuan Liu:** Investigation. **Lu-Jun Zhou:** Investigation. **Ting Liu:** Investigation. **Shuo Xu:** Investigation. **Chao-Ji Shi:** Investigation. **Hong Zhu:** Investigation. **Yong-Li Wang:** Investigation. **Jian-Yong Sun:** Investigation. **Yan Liu:** Investigation. **Wu-Chang Zhang:** Investigation. **Hai-Xia Lu:** Investigation. **Yi-Hua Wang:** Investigation. **Qiang Feng:** Investigation. **Fu-Xiang Chen:** Investigation. **Chang-Qian Wang:** Investigation. **Maurizio S. Tonetti:** Investigation. **Ya-Qin Zhu:** Funding acquisition, Supervision. **Huili Zhang:** Funding acquisition, Supervision. **Sheng-Zhong Duan:** Conceptualization, Software, Methodology, Formal analysis, Writing – review & editing, Funding acquisition, Supervision.

## Declaration of Competing Interest

The authors declare that they have no known competing financial interests or personal relationships that could have appeared to influence the work reported in this paper.

## Footnotes

Appendix A Supplementary materials include the Expanded Materials and Methods, Supplementary table (Table S1-S2) and Supplementary Figures (S1 - S11). Supplementary data to this article can be found online at https://doi.org/10.1016/j.jare.2022.03.007.
