# Supplementary material for: Roles of oral microbiota and oral-gut microbial transmission in hypertension
Source: J Adv Res. 2022 Mar 19;43:147–61. doi: 10.1016/j.jare.2022.03.007 (PMC9811375; doi:10.1016/j.jare.2022.03.007)
Supplement: Supplementary data 1 [file mmc1.docx]

**Expanded Materials and Methods**

*Sample Collection*

Participants were scheduled to collect feces samples first, and then blood samples were drawn within an hour. Saliva and subgingival plaques were collected within two hours after the blood draw. Participants who failed to collect feces first will be scheduled for another appointment until the completion of sample collection.

Feces were freshly collected by providing participants with stool collection containers in boxes with ice packs. Whole blood samples were collected in tubes with anticoagulants after at least 8 hours of fasting. Blood samples were centrifuged at 3500rpm for 15 minutes and supernatants were collected and stored at -80°C. Before collection of oral samples, participants were directed to rinse mouths and avoid eating and drinking for at least an hour. Saliva was collected in 50 ml sterile tubes (Corning, New York, USA) and preserved in Saliva DNA Preservation Solution (Huayueyang Biotech, Beijing, China). Subgingival plaques were collected using Hu-Friedy subgingival curettes and preserved in 20% glycerol.

Saliva, subgingival plaques and feces were transported to laboratory on ice packs within 2 hours after collection and stored at -80°C. A total of 133 salivary, 132 subgingival plaque and 76 fecal samples were collected and subjected to 16S rRNA sequencing.

*DNA Extraction*

DNA extraction was completed within one week after sample collection. Genomic DNA was extracted using OMEGA Soil DNA Kits (M5635-02, Omega Bio-Tek, Norcross, GA, USA) according to the manufacturer’s descriptions. The quantity and quality of extracted DNA were monitored using a NanoDrop NC2000 spectrophotometer (Thermo Fisher Scientific, Waltham, MA, USA) and agarose gel electrophoresis respectively. DNA was stored at -20°C until use.

*16S rRNA Gene Sequencing and Bioinformatic Analysis*

After DNA extraction, PCR amplification of the bacterial 16S rRNA genes V3–V4 region was performed using the forward primer 338F (5'-ACTCCTACGGGAGGCAGCA-3') and the reverse primer 806R (5'-GGACTACHVGGGTWTCTAAT-3'). Sample-specific 7-bp barcodes were incorporated into the primers for multiplex sequencing. The PCR components contained 5 μl of buffer (5×), 0.25 μl of Fast pfu DNA Polymerase (5U/μl), 2 μl (2.5 mM) of dNTPs, 1 μl (10 uM) of each Forward and Reverse primer, 1 μl of DNA Template, and 14.75 μl of ddH_2_O. Thermal cycling consisted of initial denaturation at 98 °C for 5 min, followed by 25 cycles consisting of denaturation at 98 °C for 30 s, annealing at 53 °C for 30 s, and extension at 72 °C for 45 s, with a final extension of 5 min at 72 °C. PCR amplicons were purified with Vazyme VAHTSTM DNA Clean Beads (Vazyme, Nanjing, China) and quantified using the Quant-iT PicoGreen dsDNA Assay Kit (Invitrogen, Carlsbad, CA, USA). After the individual quantification step, amplicons were pooled in equal amounts, and pair-end 2×250 bp sequencing was performed using the Illlumina MiSeq platform with MiSeq Reagent Kit v3 at Shanghai Personal Biotechnology Co., Ltd (Shanghai, China). Sequencing data were analyzed using Quantitative Insights Into Microbial Ecology (QIIME2, V.2019.4) [1]. The sequencing reads were filtered, denoised, merged, and chimera-removed by DADA2 and clustered by Vsearch (v2.13.4_linux_x86_64) and Cutadapt (v2.3) [2, 3]. The sequences were then aligned to generate phylogenetic tree using MAFFT and Fast-Tree of QIIME2 [4, 5]. Alpha and beta diversity was calculated by the diversity plugin of QIIME2. Taxonomy was assigned to ASVs using in the feature-classifier plugin of the classify-sklearn naïve Bayes taxonomy classifier against the Greengenes database [6]. A total of 8,198,803 non-chimeric sequences were generated for saliva samples, 7,802,013 for subgingival plaque samples, and 7,953,726 for fecal samples. The average non-singleton reads were 40651 for saliva, 38474 for subgingival plaque, 57091 for feces. SourceTraker analysis was used to estimate the sources of the microbiota of different sample types [7].

*Metagenomic Sequencing and Bioinformatic Analysis*

Metagenomic sequencing was performed using Whole Genome Shotgun Sequencing (Paired-end, 2 ×150 bp; insert size, 450 bp) on an Illumina NovaSeq platform at Personal Biotechnology Co., Ltd (Shanghai, China). After discard adaptors and low-quality reads [8], 2546.65 Gb high-quality pair-end reads were acquired for saliva samples, 730.5 Gb for subgingival plaque samples, and 728.05 Gb for fecal samples. The remaining reads were further filtered to remove human host DNA based on Best Match Tagger software. Reads annotation was done basing on the non-spliced sequence using Kraken2 [9], and untargeted sequences were filtered by standard procedures. Clean reads assembly was carried out by MEGAHIT, and resulting contigs > 200bp were preserved for further analysis [10]. The minimap2 was used to map high-quality reads filtered by quality control with their corresponding contig collections, and unmapped reads were reassembled and their contigs were evaluated [11]. For taxonomic annotation based on spliced sequences, contigs were aligned to the NCBI-NR database (v2019.8.12, expectation value threshold: 0.00001) by BLASTN. The taxonomical level of each contig was determined by the lowest common ancestor-based algorithm using Blast2lca (<https://github.com/emepyc/Blast2lca>).

Function annotation was performed by assigning protein sequences to the databases of Kyoto Encyclopedia of Genes and Genomes (KEGG) and Evolutionary Genealogy of Genes: Non-supervised Orthologous Groups (EggNOG). SOAPcoverage was used to calculate the abundances of contigs (https://github.com/aquaskyline/SOAPcoverage) and htseq was used to count the number of reads that aligned to the gene sequence (https://github.com/htseq/htseq). The gene abundance was calculated using transcripts per kilobase per million mapped reads as the standard of measurement.

The oral-gut transmission calculation was based on the single nucleotide variants (SNVs) analysis across metagenomic sequencing data using the metaSNV software [12]. The software could generate nucleotide diversity per sample and distance indices across samples for individual species. The distance measurements implemented by metaSNV allowed us to analyze the SNV across different samples and track specific strains based on sample-specific variants. First, 2067 species were annotated by matching the microbiota metagenomic data with the genomic database of kraken2, Bracken, and Progenomes (v2.1). These species matched with 2254 genomes in the database. Then, these genomes were used to map and generate BAM data files using Burrow-Wheeler Aligner software. The Samtools software was used to sort BAM data. Standard BAM format files were acquired after repeating sequences were filtered by Bamutil software. BAM data were used as input for metaSNV to generate the SNV diversity distance matrix. Finally, because oral-gut transmission can only occur with strains present in both oral and gut samples from the same individual, we analyzed the difference of Manhattan distance between the inter-individual (different individual) and intra-individual (the same individual). Strains that differed significantly between the inter-individual and intra-individual by Wilcoxon rank-sum test were classified into frequent oral-gut transmission category and those differed by Mann-Whitney U rank sum test were sorted into occasional oral-gut transmission category. The intra-individual distances of all transmitted strains were significantly smaller than the inter-individual distances.

*Animal Experiments*

Metronidazole (1 mg/mL), ampicillin (1 mg/mL), neomycin (1 mg/mL), gentamycin (1 mg/mL), and vancomycin (0.5 mg/mL) (Aladdin, Shanghai, China) were dissolved in sterilized water to prepare ABX. Ten-week-old male C57BL/6J mice were treated with ABX (250ul/day per mouse, replacing drinking water) for 10 days by oral gavage. Randomly selected 10 saliva samples from HTN participants (no PD) were mixed in a 50 ml sterile tube, and 10 from no HTN (no PD) participants were mixed in another tube. The mixed saliva samples were then aliquoted and suspended in an equal volume of 20% glycerol. All samples were snap-frozen in liquid nitrogen, transferred to laboratory, and stored at -80°C until use. The frozen stocks were thawed at room temperature, centrifuged at 12,000 rpm at 4°C for 3 min, resuspended in PBS, and inoculated into ABX-pretreated mice by gavage (200ul/day per mouse for 7 days). Subcutaneous minipumps (Alzet, California, USA) containing saline or angiotensin II (750 ng/kg/minute) were implanted under anesthesia with isoflurane. Before BP measurements, mice were trained to acclimatize the measurement conditions once a day for 7 days. Mice were placed in tail-cuff restrainers on a warmed surface and BP was measured by BP-2000 Blood Pressure Analysis System (Visitech Systems, North Carolina, USA). Twenty consecutive BP measurements were recorded, and the last ten readings were used for statistical analysis. Mice with different treatments were housed separately in individually ventilated cages. All animal experimental protocols were approved by the Institutional Review and Ethics Board of Shanghai Ninth People’s Hospital, Shanghai Jiao Tong University School of Medicine.

*Vasoactivity measurement*

Arterial segments (1.4 mm in length) prepared from mouse mesenteric resistance arteries were mounted on a myograph system (Danish MyoTechnology, Aarhus, Denmark) and equilibrated in Krebs solution (120 mmol/L NaCl, 25 mmol/L NaHCO3, 4.7 mmol/L KCl, 1.18 mmol/L KH2PO4, 1.18 mmol/L MgSO4, 2.5 mmol/L CaCl2, 0.026 mmol/L EDTA, and 5.5 mmol/L glucose; pH 7.4) for 60 minutes. Vascular contraction was assessed by the phenylephrine and Ang II dose response curves (10-9 to 10-5 mol/L) separately. Vascular relaxation was measured by dose response curves of acetylcholine (10-9 to 10-5 mol/L).

*Histological Analysis*

Thoracic aortas were fixed with 4% paraformaldehyde and embedded in paraffin. Cross sections (7μm) were stained with hematoxylin and eosin or 0.1% picrosirius red. All images were captured using Leica DMi8 microscope (Leica, Wetzlar, Germany) and analyzed using ImageJ software (National Institutes of Health, Bethesda, USA).

*Quantitative Real-Time-Polymerase Chain Reaction*

TRIzol reagent (Thermo Fisher Scientific, Massachusetts, USA) was used for total RNA extraction and PrimeScriptTM RT reagent Kit (Takara, Shiga, Japan) was used for reverse transcription. Quantitative reverse transcription polymerase chain reaction (QRT PCR) was performed on LightCycler480 II (Roche, Basel, Switzerland) using SYBR Green Mix (Takara, Shiga, Japan). Relative expression of each gene was determined by normalizing to 18s. The following primer sequences were used: mouse 18s, 5'-CGGCTACCACATCCAAGGAA-3' (Forward), 5'-GGGCCTCGAAAGAGTCCTGTAT-3' (Reverse); mouse Collagen-I, 5'-GCTCCTCTTAGGGGCCACT -3' (Forward), 5'-ATTGGGGACCCTTAGGCCAT-3' (Reverse); mouse ANP, 5'-CTGGGACCCCTCCGATAGAT-3' (Forward), 5'-TTCGGTACCGGAAGCTGTTG-3' (Reverse); mouse BNP, 5'-GAGTCCTTCGGTCTCAAGGC-3' (Forward), 5'-ACTTCAGTGCGTTACAGCCC-3' (Reverse).

**References**

[1] Bokulich NA, Kaehler BD, Rideout JR, Dillon M, Bolyen E, Knight R, et al. Optimizing taxonomic classification of marker-gene amplicon sequences with QIIME 2's q2-feature-classifier plugin. Microbiome. 2018;6(1):90.

[2] Callahan BJ, McMurdie PJ, Rosen MJ, Han AW, Johnson AJA, Holmes SP. DADA2: High-resolution sample inference from Illumina amplicon data. Nature methods. 2016;13(7):581-3.

[3] Edgar RC, Haas BJ, Clemente JC, Quince C, Knight R. UCHIME improves sensitivity and speed of chimera detection. Bioinformatics. 2011;27(16):2194-200.

[4] Katoh K, Misawa K, Kuma K-i, Miyata T. MAFFT: a novel method for rapid multiple sequence alignment based on fast Fourier transform. Nucleic Acids Res. 2002;30(14):3059-66.

[5] Price MN, Dehal PS, Arkin AP. FastTree: computing large minimum evolution trees with profiles instead of a distance matrix. Mol Biol Evol. 2009;26(7):1641-50.

[6] Bokulich NA, Subramanian S, Faith JJ, Gevers D, Gordon JI, Knight R, et al. Quality-filtering vastly improves diversity estimates from Illumina amplicon sequencing. Nature methods. 2013;10(1):57-9.

[7] Knights D, Kuczynski J, Charlson ES, Zaneveld J, Mozer MC, Collman RG, et al. Bayesian community-wide culture-independent microbial source tracking. Nat Methods. 2011;8(9):761-3.

[8] Chen S, Zhou Y, Chen Y, Gu J. fastp: an ultra-fast all-in-one FASTQ preprocessor. Bioinformatics. 2018;34(17):i884-i90.

[9] Wood DE, Lu J, Langmead B. Improved metagenomic analysis with Kraken 2. Genome biology. 2019;20(1):257.

[10] Li D, Liu C-M, Luo R, Sadakane K, Lam T-W. MEGAHIT: an ultra-fast single-node solution for large and complex metagenomics assembly via succinct de Bruijn graph. Bioinformatics. 2015;31(10):1674-6.

[11] Li H. Minimap2: pairwise alignment for nucleotide sequences. Bioinformatics. 2018;34(18):3094-100.

[12] Costea PI, Munch R, Coelho LP, Paoli L, Sunagawa S, Bork P. metaSNV: A tool for metagenomic strain level analysis. PLoS One. 2017;12(7):e0182392.

**Supplementary Figures**

**Fig. S1**


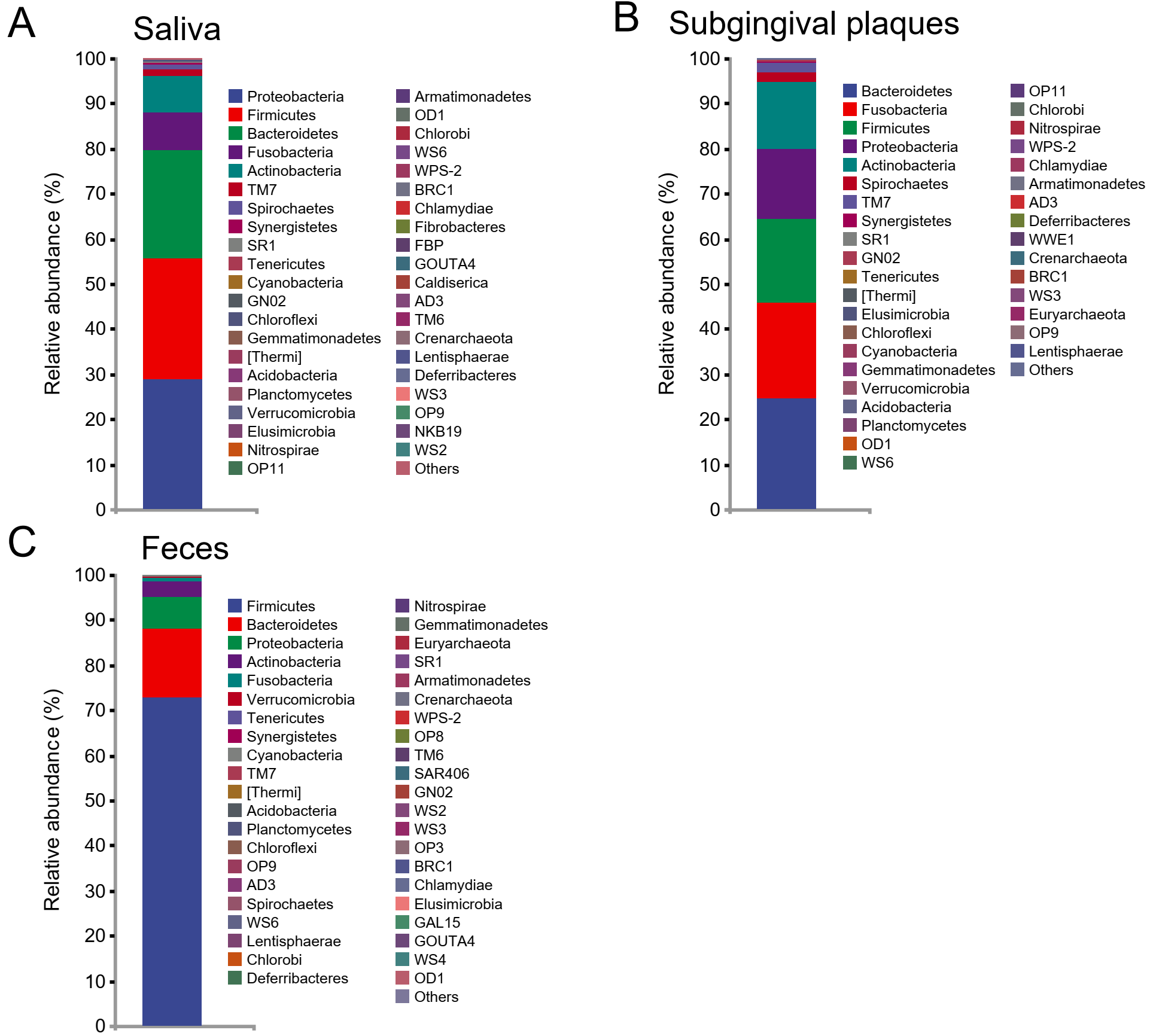


**Fig.S1 Composition of oral and gut microbiota at phylum level.** Relative abundances of microbiota of saliva (**A**), subgingival plaques (**B**), and feces (**C**) at phylum level. n=133 for A, 132 for B, and 76 for C.

**Fig. S2**


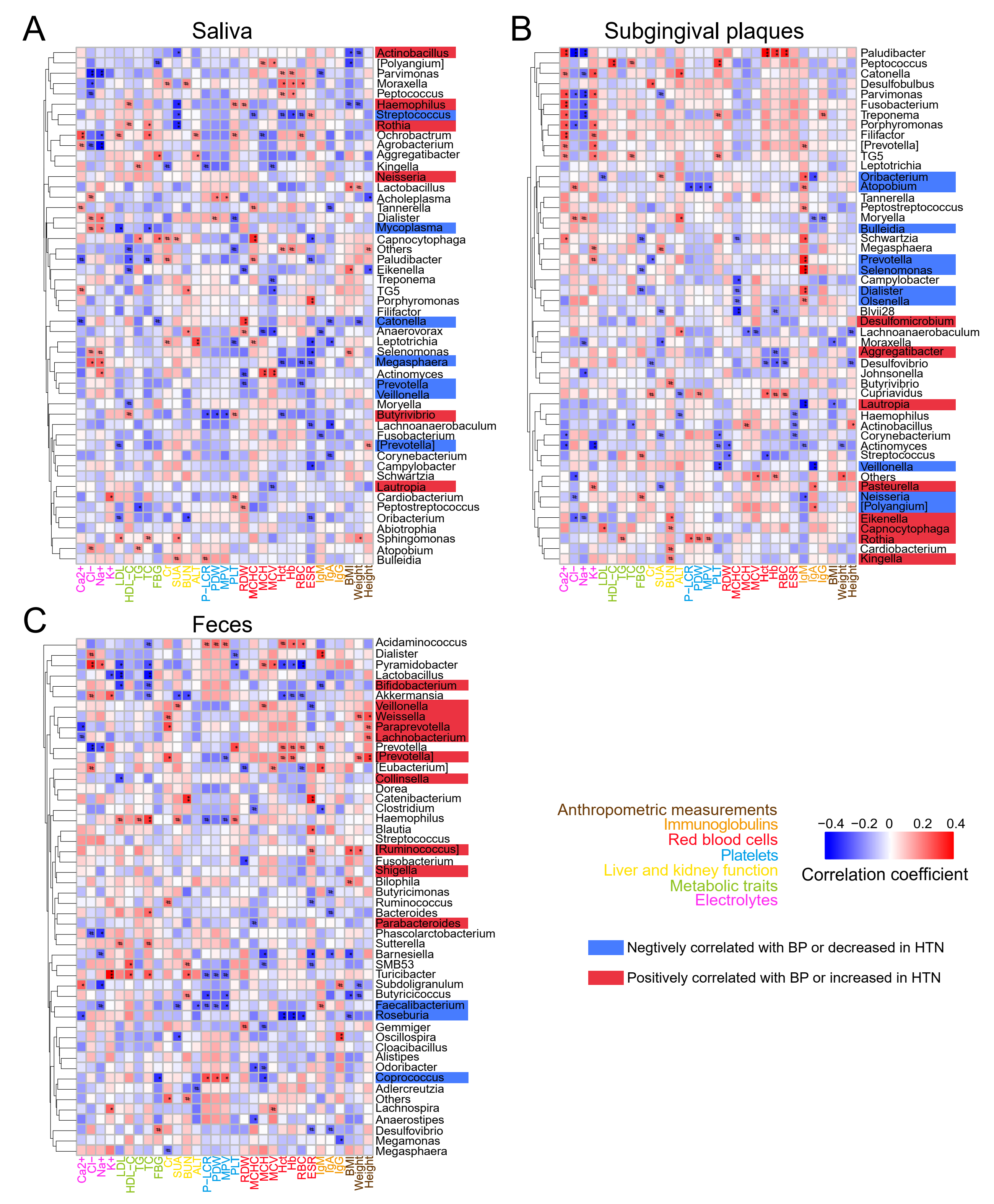


**Fig.S2 Associations between oral/gut microbiota and clinical parameters in participants with HTN.** Clustering heatmaps of Spearman’s correlation coefficients between clinical parameters (anthropometric measurements, red blood cells, platelets, liver and kidney function, metabolic traits, and electrolytes) and relative abundances of the top 50 genera in microbiota of saliva (A), subgingival plaques (B), and feces (C). n=77 for saliva and subgingival plaques, and 76 for feces. Ca2+: calcium, Cl-: chlorine, Na+: sodium, K+: potassium, LDL: low-density lipoprotein cholesterol, HDL-C: high density lipoprotein cholesterol, TG: triglyceride, TC: total cholesterol, FBG: fasting blood glucose, Cr: creatinine, SUA: serum uric acid, BUN: blood urea nitrogen, ALT: alanine aminotransferase, P-LCR: platelet-large cell ratio, PDW: platelet distribution width, MPV: mean platelet volume, PLT: platelet, RDW: red blood cell distribution width, MCHC: mean corpuscular hemoglobin concentration, MCH: mean corpuscular hemoglobin, MCV: mean corpuscular volume, Hct: hematocrit, Hb: hemoglobin, RBC: red blood cells, ESR: Erythrocyte sedimentation rate, Ig: Immunoglobulin, BMI: body mass index. #p(FDR) < 0.1, *p(FDR) < 0.05, **p(FDR) < 0.01, ***p(FDR) < 0.001.

**Fig. S3**


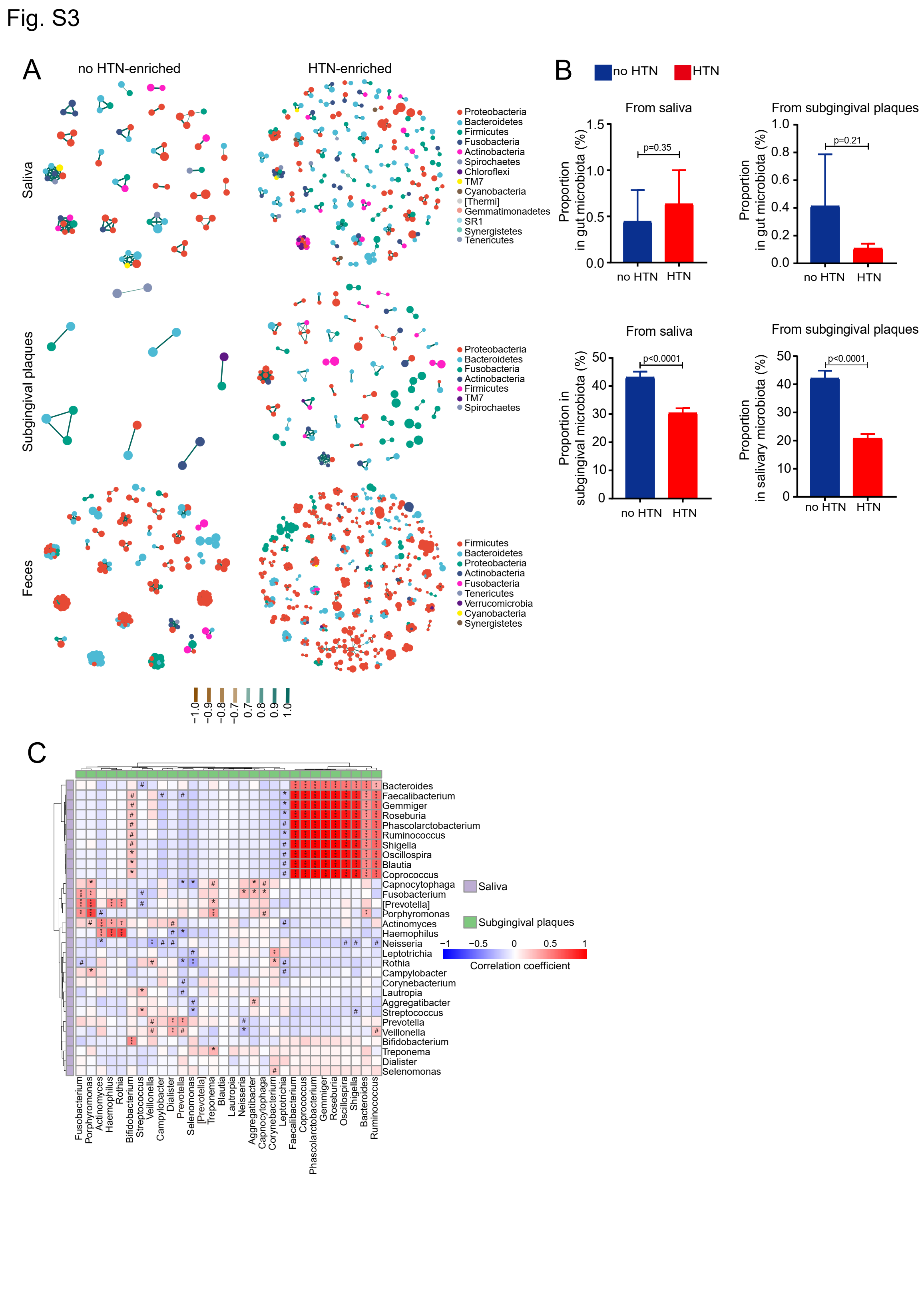


**Fig.S3 Communications between oral and gut microbiota in participants with or without HTN. A.** Microbial abundance co-correlation networks within salivary, subgingival, or gut microbiota at phylum level. Brown and green lines indicate negative and positive correlations between phyla respectively. Node size reflects relative abundance. **B.** SourceTracker analysis to estimate microbial communications from oral cavity to gut and within oral cavity. **C.** Heatmap of Spearman’s correlation coefficients between relative abundances of shared genera in salivary microbiota and those in subgingival microbiota. n=39:94 for saliva, 39:93 for subgingival plaques, and 24:52 for feces in A and C. n=132 for saliva and subgingival plaques in B. #p(FDR) < 0.1, *p(FDR) < 0.05, **p(FDR) < 0.01, ***p(FDR) < 0.001.

**Fig. S4**


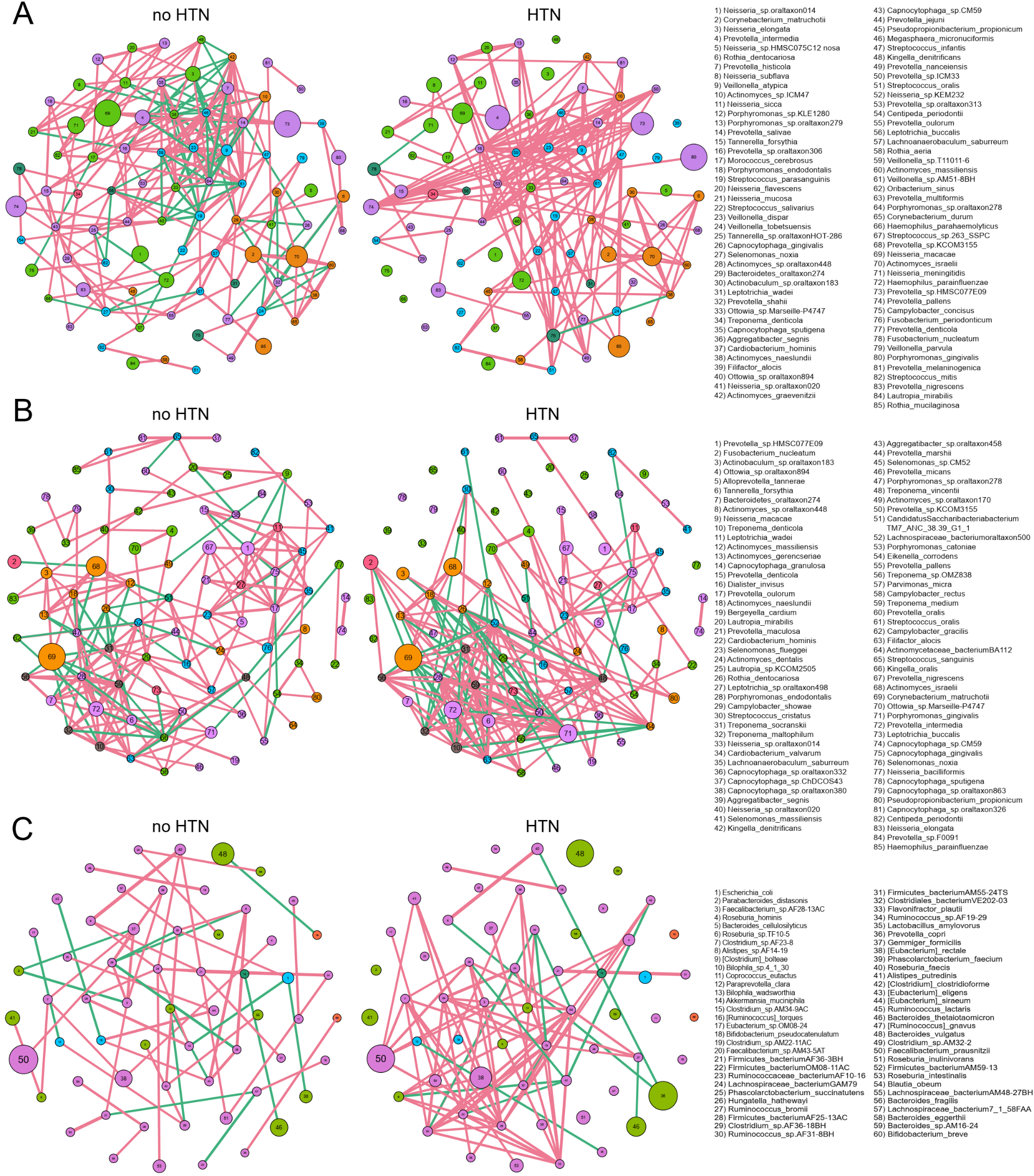


**Fig.S4 Co-correlation networks of predominant species in participants with or without HTN.** Abundance co-correlation networks of predominant species within salivary (**A**), subgingival (**B**), and gut microbiota (**C**) of no HTN and HTN groups. The size of circles indicates the abundance of species. All microbiota was analyzed using metagenomic sequencing. Pink and green lines indicate negative and positive correlations respectively. The depicted predominant species were those among the top 100 species and with r>0.6 and p<0.01 by Spearman’s correlation analysis. n=24:36 for all samples.

**Fig. S5**


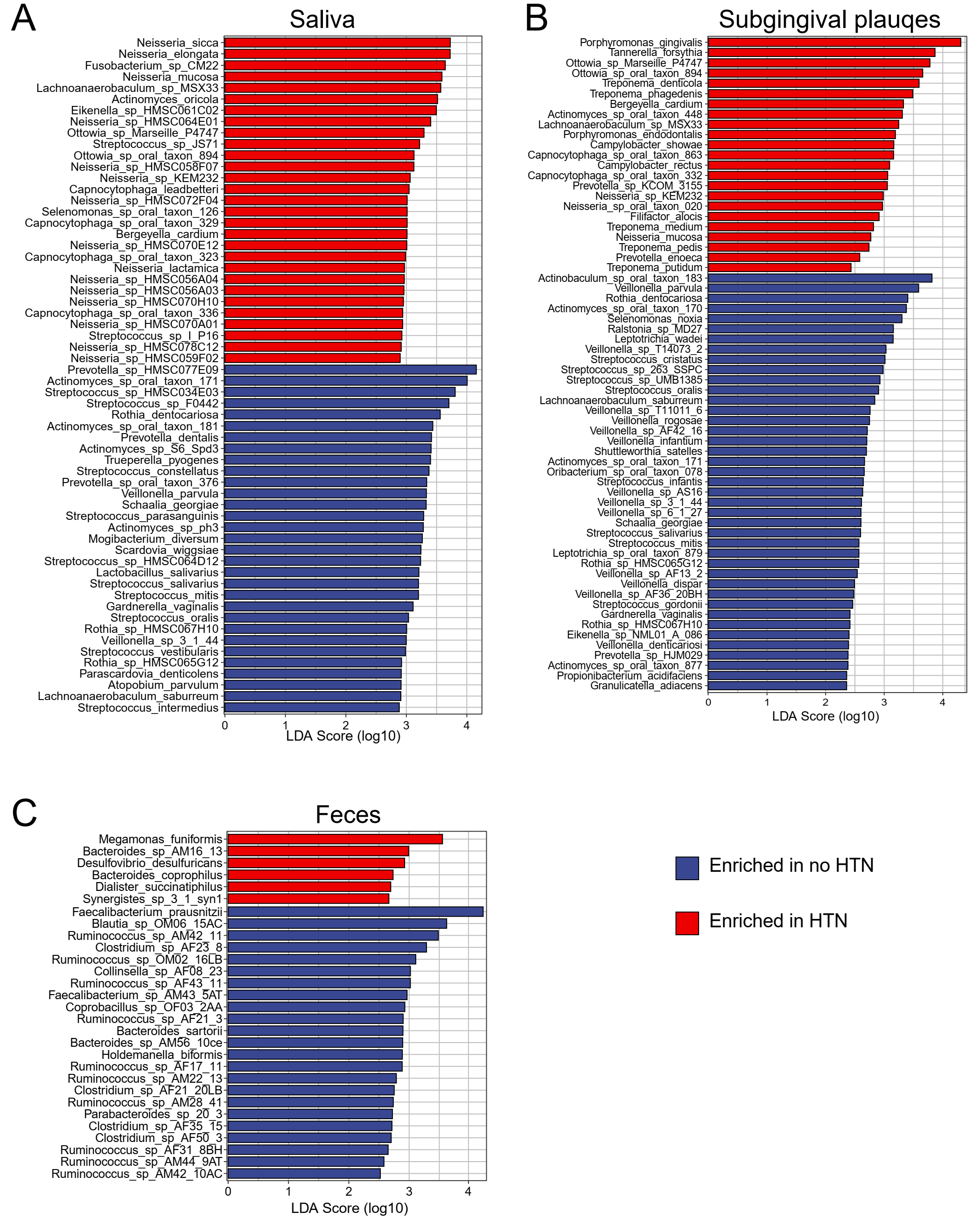


**Fig.S5 Species enrichment analyzed by metagenomic sequencing in participants with or without HTN.** Linear discriminant analysis effect size (LEfSe) revealing differentially enriched species between no HTN and HTN in saliva (**A**), subgingival plaques (**B**), and feces (**C**). n=24:36 for all samples.

**Fig. S6**


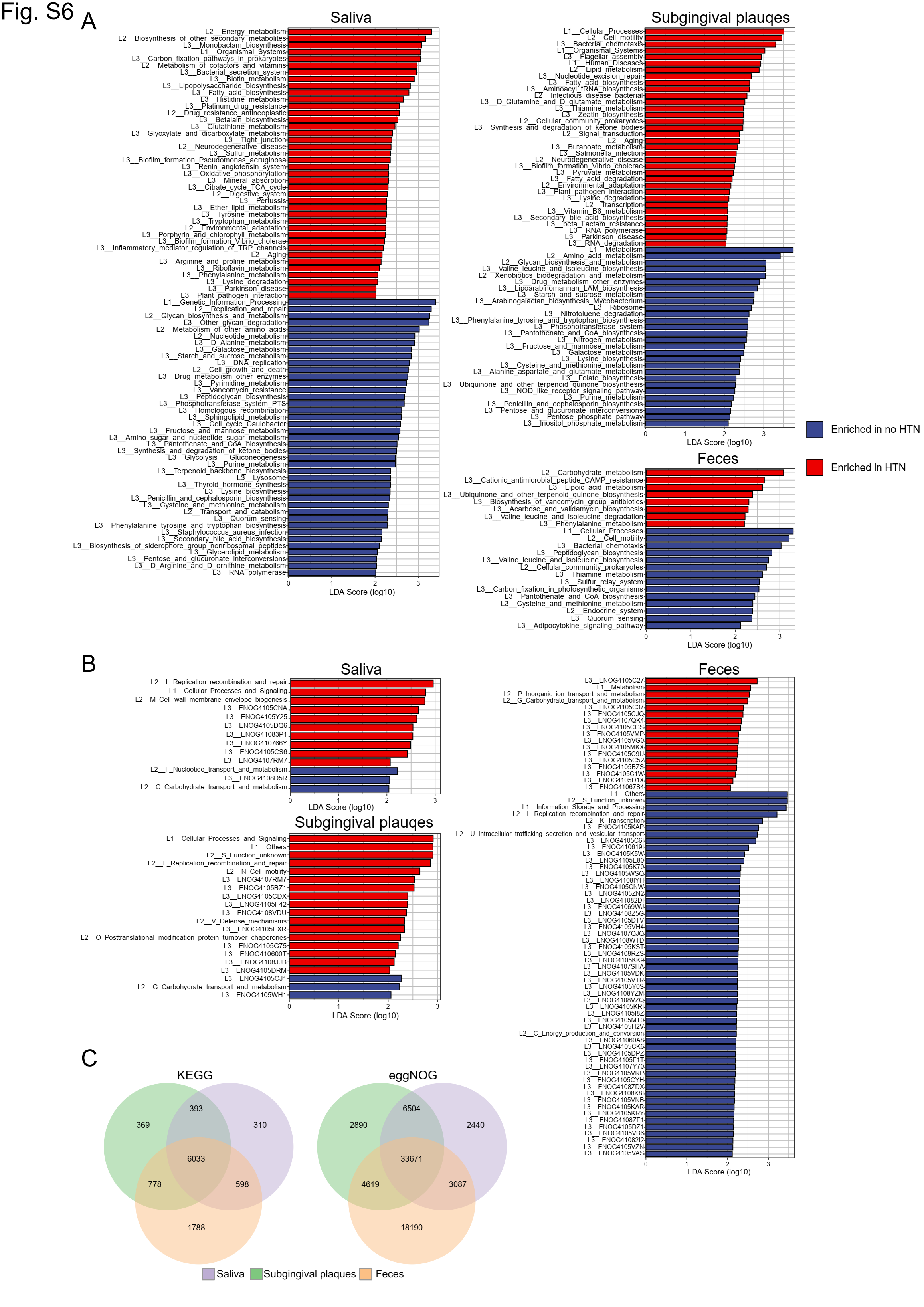


**Fig.S6 Functional enrichment of KEGG orthologous genes and eggnog orthologous genes in no HTN or HTN. A-B.** LEfSe of KEGG orthologous genes (A) or eggNOG orthologous genes (B) enriched in no HTN or HTN. **C.** Venn diagrams showing overlaps of KEGG orthologous genes and eggNOG orthologous genes among saliva, subgingival plaques, and feces. All microbiota was analyzed using metagenomic sequencing. n=24:36 for all samples.

**Fig. S7**


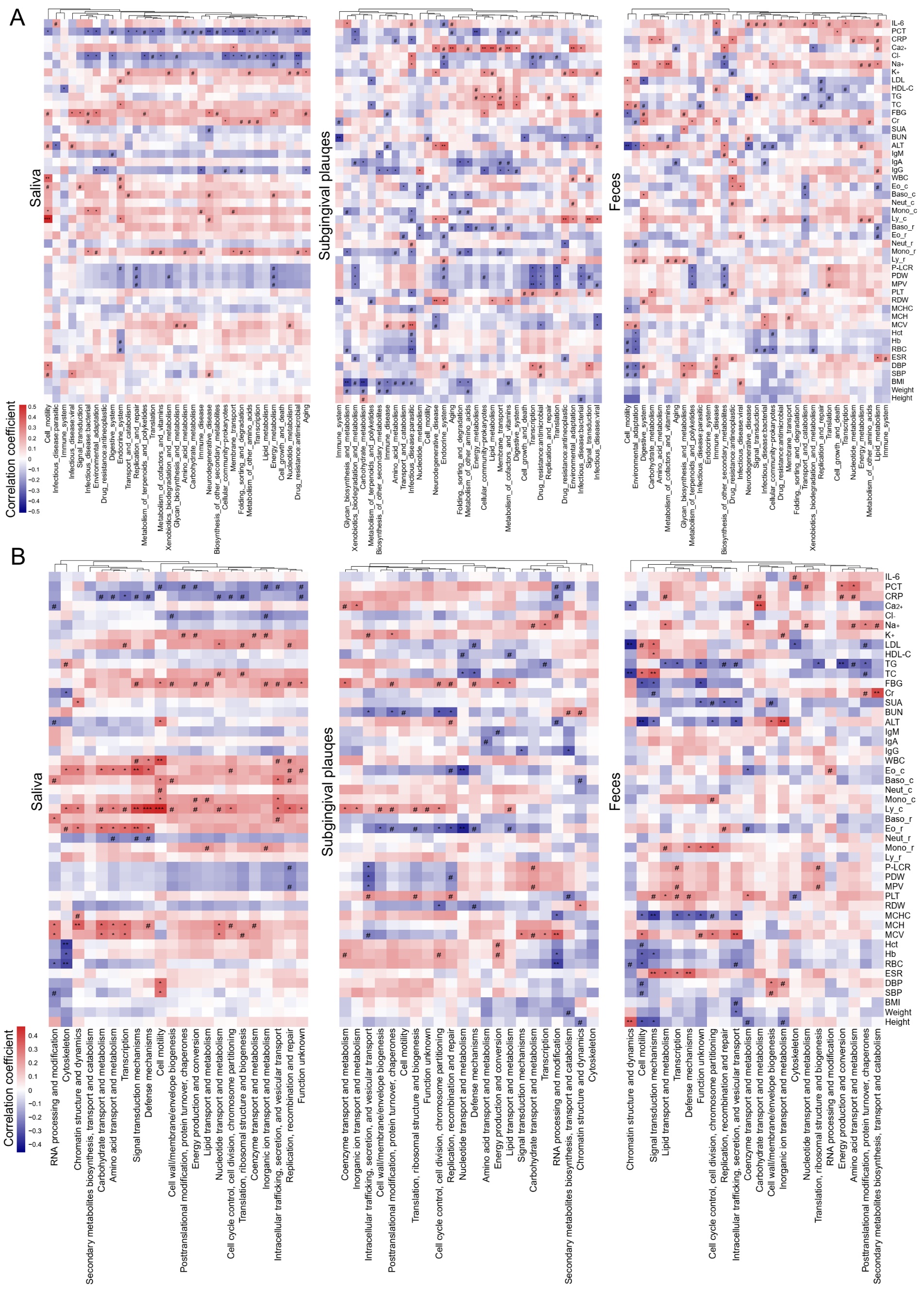


**Fig.S7 Associations between clinical parameters and microbial functional modules.** Heatmaps of Spearman’s correlation coefficients between clinical parameters and microbial functional modules annotated by KEGG (**A**) or eggNOG (**B**). All microbiota was analyzed using metagenomic sequencing. n=60 for all samples. #p(FDR) < 0.1, *p(FDR) < 0.05, **p(FDR) < 0.01, ***p(FDR) < 0.001.

**Fig. S8**


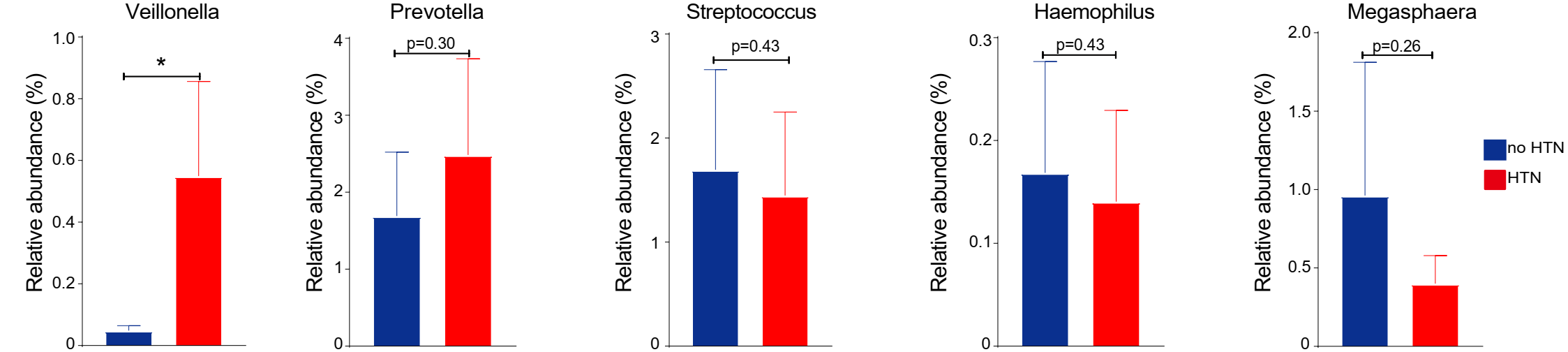


**Fig.S8 Comparisons of relative abundances of the 5 oral-gut-transmitting genera in feces between no HTN and HTN participants of the cross-sectional study.** All microbiota was analyzed using 16S rRNA gene sequencing. n= 24:52. Student’s *t* test was used for statistical analysis. *p < 0.05.

**Fig. S9**


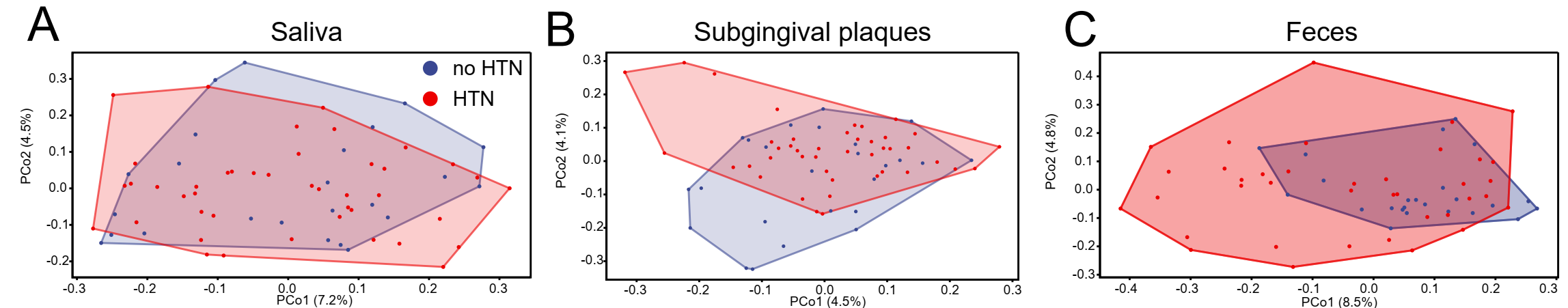


**Fig.S9 PCoA of oral and gut microbiota in participants with or without HTN after 6 months.** PCoA of microbiota of saliva (**A**), subgingival plaques (**B**), and feces (**C**) in participants with or without HTN after a 6-month follow-up. All microbiota was analyzed using 16S rRNA gene sequencing. n=24:43 for saliva, 25:43 for subgingival plaques, and 24:38 for feces.

**Fig. S10**


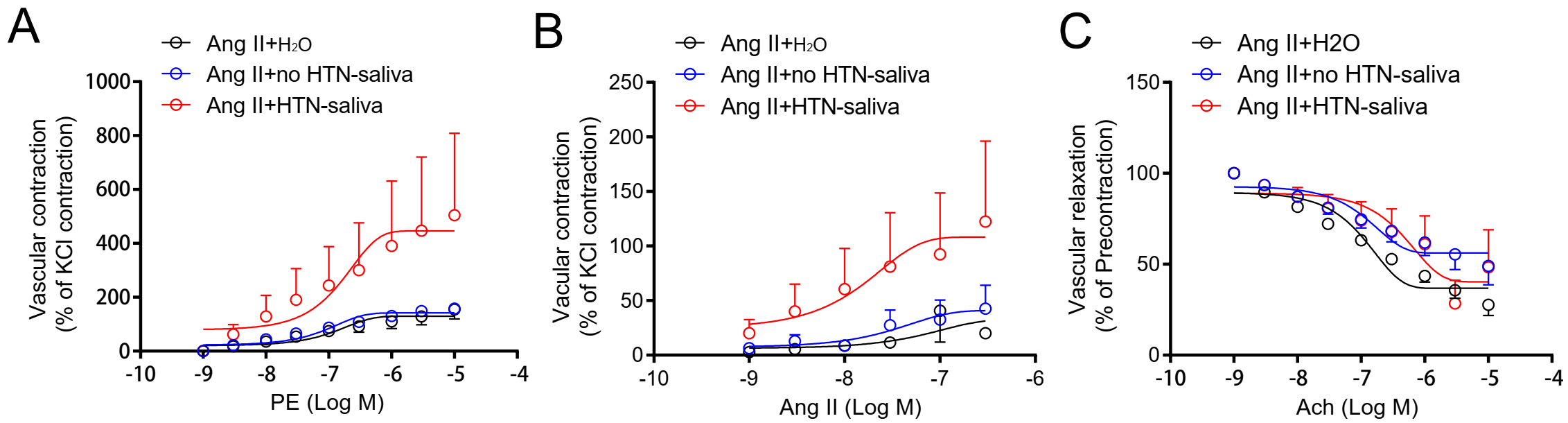


**Fig.S10 Effects of oral microbiota on vasoactivity in mice.** The contractility of mesenteric arteries was measured by phenylephrine (A) and angiotensin II (B) doseresponse curves. The relaxation of mesenteric arteries was measured by acetylcholine (C) dose-response curves. n=4:4:3 for H2O+Ang II vs no HTN-saliva+Ang II vs HTNsaliva+Ang II.

**Fig. S11**


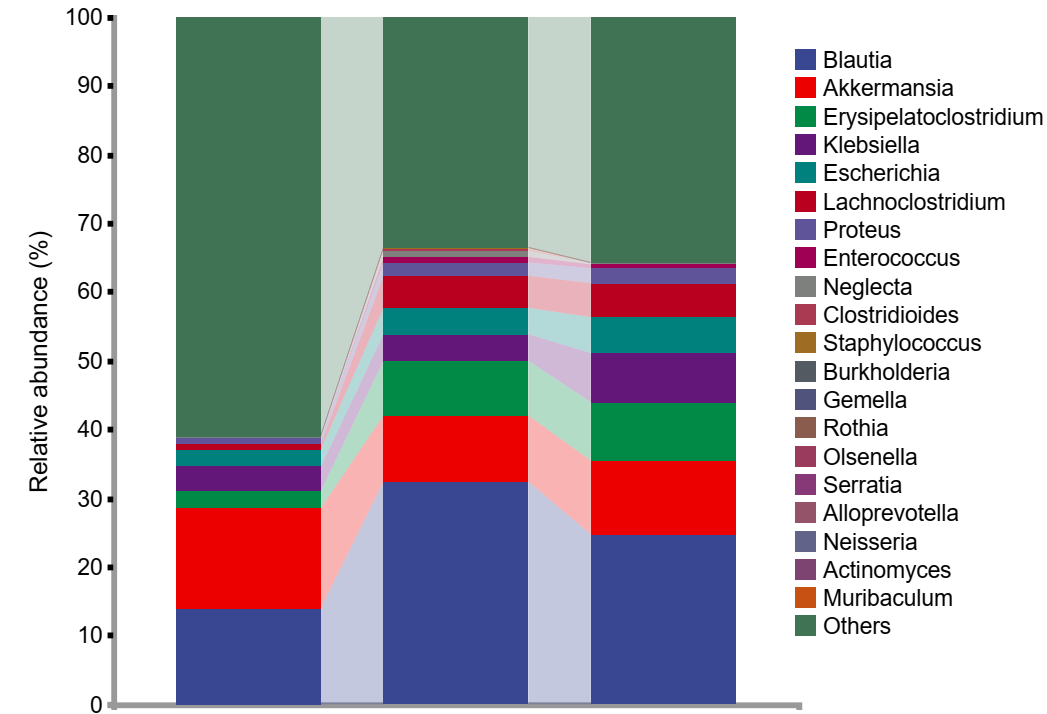


**Fig.S11 Composition of gut microbiota of mice treated with ABX only at genus level.** Stacked bar plots show the top 20 gut microbial genera in mice treated with ABX only. None of the 5 oral-gut transmitting genera (*Veillonella*, *Streptococcus*, *Prevotella*, *Haemophilus*, and *Megasphaera*) was detected after ABX treatment. n=3.
