# Supplementary material for: Roles of oral microbiota and oral-gut microbial transmission in hypertension
Source: J Adv Res. 2022 Mar 19;43:147–61. doi: 10.1016/j.jare.2022.03.007 (PMC9811375; doi:10.1016/j.jare.2022.03.007)
Supplement: Supplementary data 3 [file mmc3.docx]

**Table S2 Results of Adonis analysis**

| **Feces** |  |  |  |  |  |  |
| --- | --- | --- | --- | --- | --- | --- |
| **Items** | **Df** | **SumsOfSqs** | **MeanSqs** | **F.Model** | **R2** | **Pr (>F)** |
| Diabetes mellitus VS non-Diabetes mellitus | 1 | 0.378554 | 0.378554 | 1.05315 | 0.011441 | 0.288 |
| Antihypertensive treatment VS non-Antihypertensive treatment | 1 | 0.334987 | 0.334987 | 0.930706 | 0.010124 | 0.708 |
| **Subgingival plaques** |  |  |  |  |  |  |
| **Items** | **Df** | **SumsOfSqs** | **MeanSqs** | **F.Model** | **R2** | **Pr (>F)** |
| Diabetes mellitus VS non-Diabetes mellitus | 1 | 0.380237 | 0.380237 | 0.958925 | 0.018818 | 0.544 |
| Antihypertensive treatment VS non-Antihypertensive treatment | 1 | 0.462768 | 0.462768 | 1.171939 | 0.022902 | 0.17 |
| **Saliva** |  |  |  |  |  |  |
| **Items** | **Df** | **SumsOfSqs** | **MeanSqs** | **F.Model** | **R2** | **Pr (>F)** |
| Diabetes mellitus VS non-Diabetes mellitus | 1 | 0.379514 | 0.379514 | 1.023785 | 0.011006 | 0.361 |
| Antihypertensive treatment VS non-Antihypertensive treatment | 1 | 0.40474 | 0.40474 | 1.092395 | 0.011862 | 0.276 |
