# Supplementary material for: Roles of oral microbiota and oral-gut microbial transmission in hypertension
Source: J Adv Res. 2022 Mar 19;43:147–61. doi: 10.1016/j.jare.2022.03.007 (PMC9811375; doi:10.1016/j.jare.2022.03.007)
Supplement: Supplementary data 4 [file mmc4.docx]

**Data for reviewers**


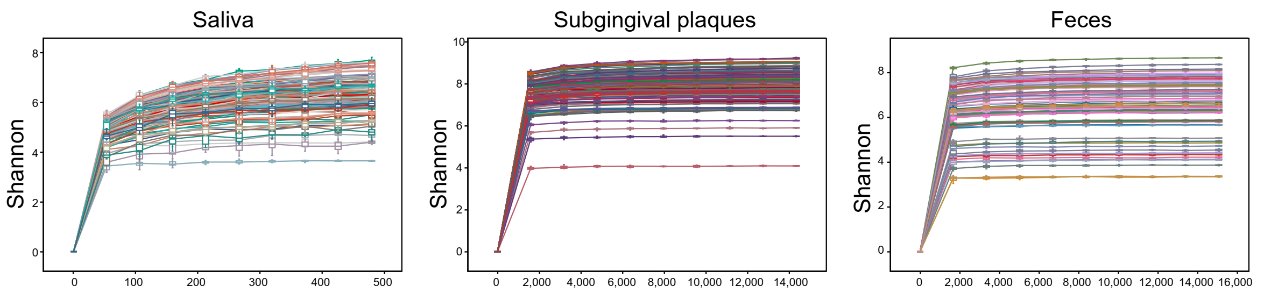


**Rarefaction curves of oral and gut microbiota in each sample.** Shannon rarefaction curves of salivary, subgingival, and fecal microbiota of each individual sample are shown. All microbiota was analyzed using 16S rRNA gene sequencing. n=133 for saliva, 132 for subgingival plaques, and 76 for feces.
